# Structural Basis for Rab1 De-AMPylation by the *Legionella pneumophila* Effector SidD

**DOI:** 10.1371/journal.ppat.1003382

**Published:** 2013-05-16

**Authors:** Yang Chen, Igor Tascón, M. Ramona Neunuebel, Chiara Pallara, Jacqueline Brady, Lisa N. Kinch, Juan Fernández-Recio, Adriana L. Rojas, Matthias P. Machner, Aitor Hierro

**Affiliations:** 1 Cell Biology and Metabolism Program, *Eunice Kennedy Shriver* National Institute of Child Health and Human Development, National Institutes of Health, Bethesda, Maryland, United States of America; 2 Structural Biology Unit, CIC bioGUNE, Bizkaia Technology Park, Derio, Spain; 3 Joint BSC-IRB research program in Computational Biology, Barcelona Supercomputing Center, Barcelona, Spain; 4 University of Texas Southwestern Medical Center, Dallas, Texas, United States of America; 5 IKERBASQUE, Basque Foundation for Science, Bilbao, Spain; Tufts University School of Medicine, United States of America

## Abstract

The covalent attachment of adenosine monophosphate (AMP) to proteins, a process called AMPylation (adenylylation), has recently emerged as a novel theme in microbial pathogenesis. Although several AMPylating enzymes have been characterized, the only known virulence protein with de-AMPylation activity is SidD from the human pathogen *Legionella pneumophila*. SidD de-AMPylates mammalian Rab1, a small GTPase involved in secretory vesicle transport, thereby targeting the host protein for inactivation. The molecular mechanisms underlying Rab1 recognition and de-AMPylation by SidD are unclear. Here, we report the crystal structure of the catalytic region of SidD at 1.6 Å resolution. The structure reveals a phosphatase-like fold with additional structural elements not present in generic PP2C-type phosphatases. The catalytic pocket contains a binuclear metal-binding site characteristic of hydrolytic metalloenzymes, with strong dependency on magnesium ions. Subsequent docking and molecular dynamics simulations between SidD and Rab1 revealed the interface contacts and the energetic contribution of key residues to the interaction. In conjunction with an extensive structure-based mutational analysis, we provide *in vivo* and *in vitro* evidence for a remarkable adaptation of SidD to its host cell target Rab1 which explains how this effector confers specificity to the reaction it catalyses.

## Author Summary

The covalent attachment of adenosine monophosphate (AMP) to proteins, a process called AMPylation (adenylylation), has recently emerged as a novel theme in microbial pathogenesis. While AMPylases from various pathogenic microorganisms have recently been characterized, the only virulence protein with de-AMPylation activity known to date is the *Legionella pneumophila* effector SidD which catalyzes AMP removal from the host GTPase Rab1. Thus, both AMPylation and de-AMPylation constitute a novel catalytic mechanism to precisely control the function and membrane dynamics of a host Rab GTPase. In spite of this pivotal role, the molecular mechanism of AMP removal and the structural determinants for Rab1 recognition by SidD have remained largely unexplored. Here, we present the crystal structure of the de-adenylylation domain of SidD and reveal the catalytic mechanism of Rab1 de-adenylylation. Surprisingly, the structure of SidD is not related to the other known enzyme with de-AMPylation activity, the *Escherichia coli* GS-ATase. Instead, the catalitic domain of SidD is remarkably similar to that of the metal-dependent protein phosphatases (PPMs), however with distinctive structural features to distinguish AMPylated Rab1 from similarly modified substrates. Importantly, we provide a model for the SidD-Rab1 complex which sheds light into the specific details of substrate recognition and catalysis by this virulence factor.

## Introduction

Microbial pathogens have developed a diverse spectrum of mechanisms to manipulate the human host and cause disease. Many bacterial proteins post-translationally modify host factors in order to alter their function. The covalent attachment of adenosine monophsophate (AMP) to threonine or tyrosine side chains within proteins, a process known as AMPylation (adenylylation), was discovered more than 40 years ago in the *Escherichia coli* protein glutamine synthetase adenylyl transferase (GS-ATase) which regulates the enzyme glutamine synthetase through reversible AMPylation [1]. This post-translational modification recently re-emerged with the discovery of several virulence proteins from Gram-negative bacteria such as *Vibrio parahaemolyticus*, *Histophilus somni*, and *Legionella pneumophila* that AMPylate host proteins [2], [3], [4]. Surprisingly, each of these AMPylators was shown to target host cell GTPases of the Rho or Rab family. VopS from *V. parahaemolyticus* and IbpA from *H. somni* covalently modify Rho GTPases such as Cdc42 and Rac1 with AMP, thereby causing a collapse of the host cell actin cytoskeleton resulting in cell rounding [2], [3]. In contrast, SidM (DrrA) from *L. penumophila* AMPylates host cell Rab GTPases [4] thereby exploiting intracellular vesicle trafficking routes.

The finding that host cell GTPases are a preferred target of bacterial AMPylators can be attributed to the fundamental role these proteins play in all eukaryotic cells. Rab proteins regulate virtually all aspects of vesicle transport [5], [6]. They function as molecular switches that cycle between an inactive GDP-bound state with predominantly cytosolic distribution and an active GTP-bound form that is associated with organelle membranes [7], [8], [9]. Rab activation requires a guanine nucleotide exchange factor (GEF) which promotes replacement of GDP with GTP to enhance the recruitment of downstream ligands, whereas Rab inactivation requires GTPase-activating proteins (GAPs) that stimulate the hydrolysis of GTP to GDP. Inactive GDP-bound Rabs are subsequently extracted from the membrane by a GDP dissociation inhibitor (GDI) and maintained in the cytosol for the next recruitment cycle.

The opportunistic pathogen *L. pneumophila*, the causative agent of a severe pneumonia known as Legionnaires' disease, subverts membrane dynamics of the host cell by intercepting and modulating Rab1 [10], [11], [12], [13], the regulator of endoplasmic reticulum (ER) to Golgi vesicle transport. The organism infects human alveolar macrophages and multiplies within a specialized compartment called the *Legionella*-containing vacuole (LCV). To ensure intracellular survival, *L. pneumophila* uses a specialized translocation machine known as the Dot/Icm type IV secretion system (T4SS) which mediates the delivery of over 200 proteins, termed effectors, from its own cytosol into the host cytoplasm [14]. The effector SidM (DrrA) binds phosphatidylinositol 4-phosphate present in the LCV membrane [15] and exhibits GEF as well as GDF activity towards host cell Rab1 [16], [17], thereby accumulating active GTP-Rab1 on the LCV surface. SidM then AMPylates tyrosine-77 located in the switch II region of Rab1 (Y77_Rab1_) [4]. The bulky AMP moiety is believed to sterically interfere with the ability of Rab1 to interact with downstream ligands, most importantly GAPs such as the *L. pneumophila* Rab1GAP LepB, thereby making Rab1 insensitive to inactivation and maximizing its accumulation on the LCV. Notably, activated AMP-Rab1 is gradually removed from the compartment in a process that depends on the *L. pneumophila* effector protein SidD [18], [19]. SidD is delivered into the host cell later than the AMPylase SidM and catalyzes AMP removal from Rab1, a reaction referred to as de-AMPylation (or de-adenylylation). Once Rab1 has been de-AMPylated, it becomes accessible to binding and inactivation by LepB and subsequent GDI-mediated extraction from LCV membranes [18], [19]. The ability of *L. pneumophila* to regulate Rab1 membrane cycling through AMPylation and de-AMPylation provides a precedent for how reversible post-translational modification may be used by pathogens to precisely control the function of small Rab GTPases within host cells.

To our knowledge, *L. pneumophila* SidD and the N-terminal domain (AT-N) of the *E. coli* GS-ATase are the only known enzymes with de-AMPylation activity, yet the reactions they catalyze differ significantly: While AMP removal performed by AT-N is strictly dependent on the presence of orthophosphate and produces ADP [20], Rab1 de-AMPylation by SidD is phosphate-independent and generates AMP [18], indicative of two fundamentally different mechanisms of de-AMPylation. SidD lacks any obvious sequence homology with the AT-N or other known proteins, although fold recognition analysis of the N-terminal portion of SidD predicted limited resemblance with members of the metal-dependent protein phosphatase (PPM) family. The conserved aspartate residues at position 92 and 110, which are crucial for the activity of other phosphatases, also contribute structurally or chemically to SidD's catalysis [19]. Nonetheless, the molecular mechanism of AMP removal and the structural determinants for Rab1 recognition by SidD have remained largely unexplored.

In this study, we use a multidisciplinary approach to characterize the structural and molecular details that determine substrate recognition and catalysis by SidD. We discover a unique mechanism by which SidD identifies AMPylated Rab1 but not Rho GTPases and that it performs de-AMPylation but not the chemically related de-phosphocholination reaction.

## Results

### SidD is composed of two functionally distinct regions

**Figure 1:**
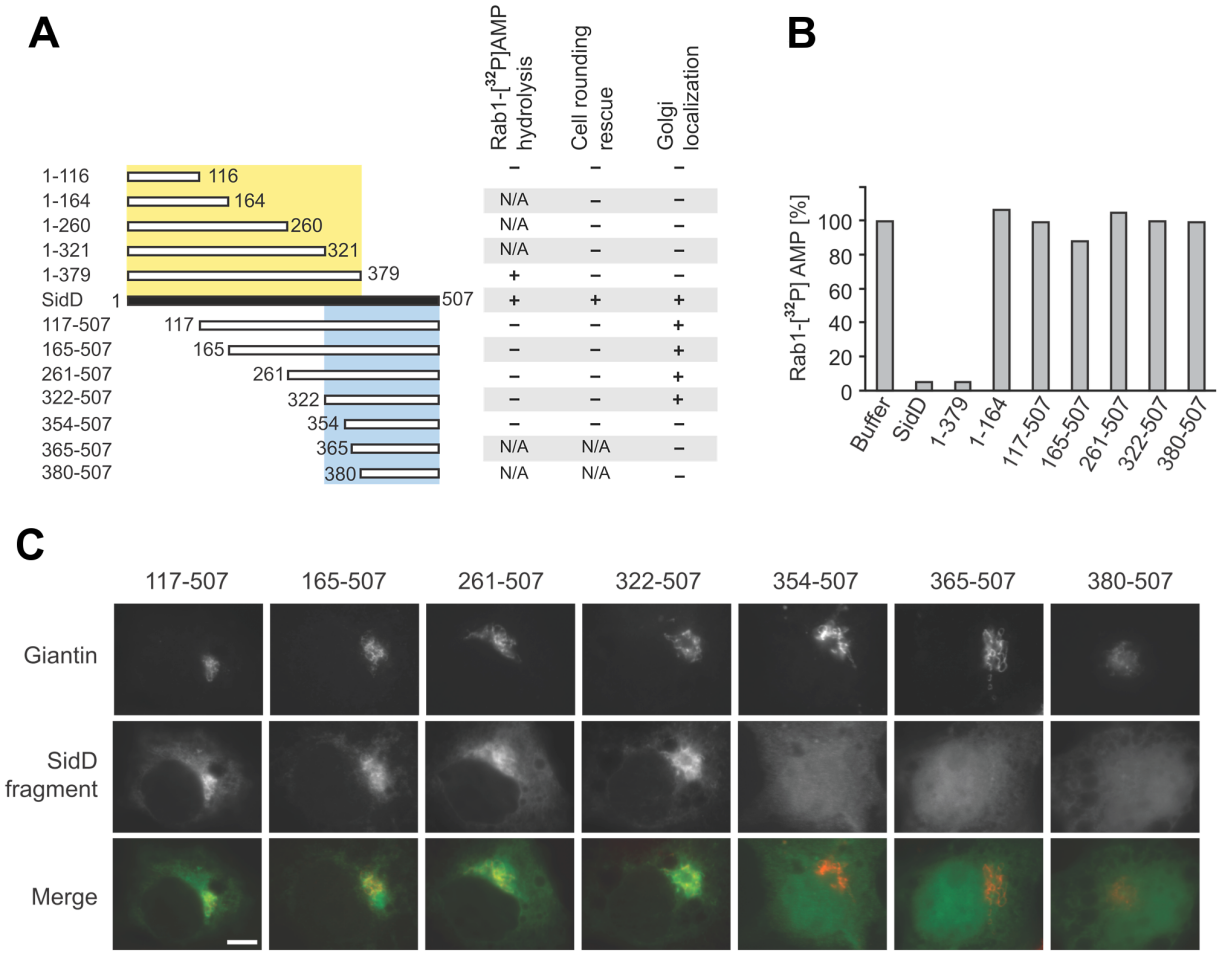
SidD is composed of an N-terminal catalytic domain and a membrane-targeting region. (A) Schematic representation of SidD and its variants used in this study (numbers indicate amino acid positions). The chart summarizes the data presented in (B), (C), as well as the results from the cell rounding rescue assay (not shown) indicating positive (+) or negative (−) outcomes. The region highlighted in yellow (residue 1 to 379) is required for de-AMPylation activity of SidD whereas the region between amino acid 322 and 507 (blue) mediates Golgi localization of SidD. The overlapping region of 57 residues is required for both activities. Only the full-length protein showed activity in each of the three assays. (B) De-AMPylation activity of SidD variants. [^32^P]AMP-Rab1 (2 µM) was incubated with SidD variants at a molar ratio of 1∶100. Samples were taken after 30 min and the amount of [^32^P]AMP-Rab1 was determined by scintillation counting. The graph is a representation of two independent experiments with almost identical outcomes. (C) The C-terminal region targets SidD to the Golgi compartment. Transiently transfected COS1 cells producing GFP-SidD variants (middle panels) were fixed and stained for the Golgi marker giantin (top panels). The merged images (bottom panels) show SidD variants in green and giantin in red. Scale bar represents 1 µm.

The primary sequence of SidD consisting of 507 amino acids shows no homology to other proteins. Thus, it was unclear which part of SidD possessed de-AMPylation activity and if the protein potentially exhibited additional functions. Guided by secondary structure predictions we created N- or C-terminally truncated variants of SidD, purified them from *E. coli*, and tested their ability to catalyze removal of radiolabeled [α^32^P]AMP from Rab1 *in vitro* (Figure 1A, B). We found that none of the C-terminal fragments and only the longest N-terminal variant spanning amino acid 1 to 379 (SidD_1–379_) displayed catalytic activity comparable to the full length protein. We also noticed that several of the shorter variants (SidD_1–321_, SidD_1–260_, or SidD_1–164_) were produced either as insoluble or unstable proteins in E. coli (data not shown), suggesting that proper folding of these fragments was compromised by the truncations.

To reduce folding or stability problems that might occur during protein production in *E. coli*, we employed a mammalian cell-based assay to analyze the de-AMPylation activity of the SidD variants within their host environment. We previously described that production of SidM in transiently transfected COS1 cells causes Golgi fragmentation and subsequent cell rounding and that this phenomenon can be partially repressed by simultaneously producing SidD in the same cell [18], consistent with the fact that SidD's de-AMPylation activity antagonizes SidM's AMPylation activity. When analyzing GFP-tagged SidD variants in this rescue assay we found that none of the truncated proteins was capable of efficiently preventing SidM-induced COS1 cell rounding (Figure 1A), not even SidD_1–379_, the longest N-terminal fragment that exhibited full Rab1 de-AMPylation activity *in vitro* (Figure 1B). The failure to rescue cell rounding was not due to the absence or instability of the truncated SidD variants (Figure S1). Rather, we noticed a difference in the intracellular localization pattern of some SidD fragments compared to that of full length GFP-SidD which, as we reported earlier, colocalizes with marker proteins of the Golgi and trans-Golgi network [18]. Upon closer examination, we found that only SidD variants containing the C-terminal 185 residues (amino acid 322 to 507) displayed colocalization with the Golgi marker giantin similar to that of full length SidD (Figure 1C). None of the N-terminal fragments were enriched at the Golgi but instead showed a predominantly cytosolic distribution pattern. Thus, the C-terminal region spanning amino acid 322–507 possessed the ability to target SidD to the Golgi by interacting with a yet unknown factor on this compartment, and failure of GFP-SidD_1–379_ to properly localize to the correct target organelle may explain the inability of this catalytically active SidD fragment to rescue SidM-mediated cell rounding (Figure 1A). Based on these results we divided SidD into two functional regions: an N-terminal domain with de-AMPylation activity (aa 1–379) and a C-terminal targeting region (aa 322–507).

### Overall structure of SidD's de-AMPylation domain

**Figure 2:**
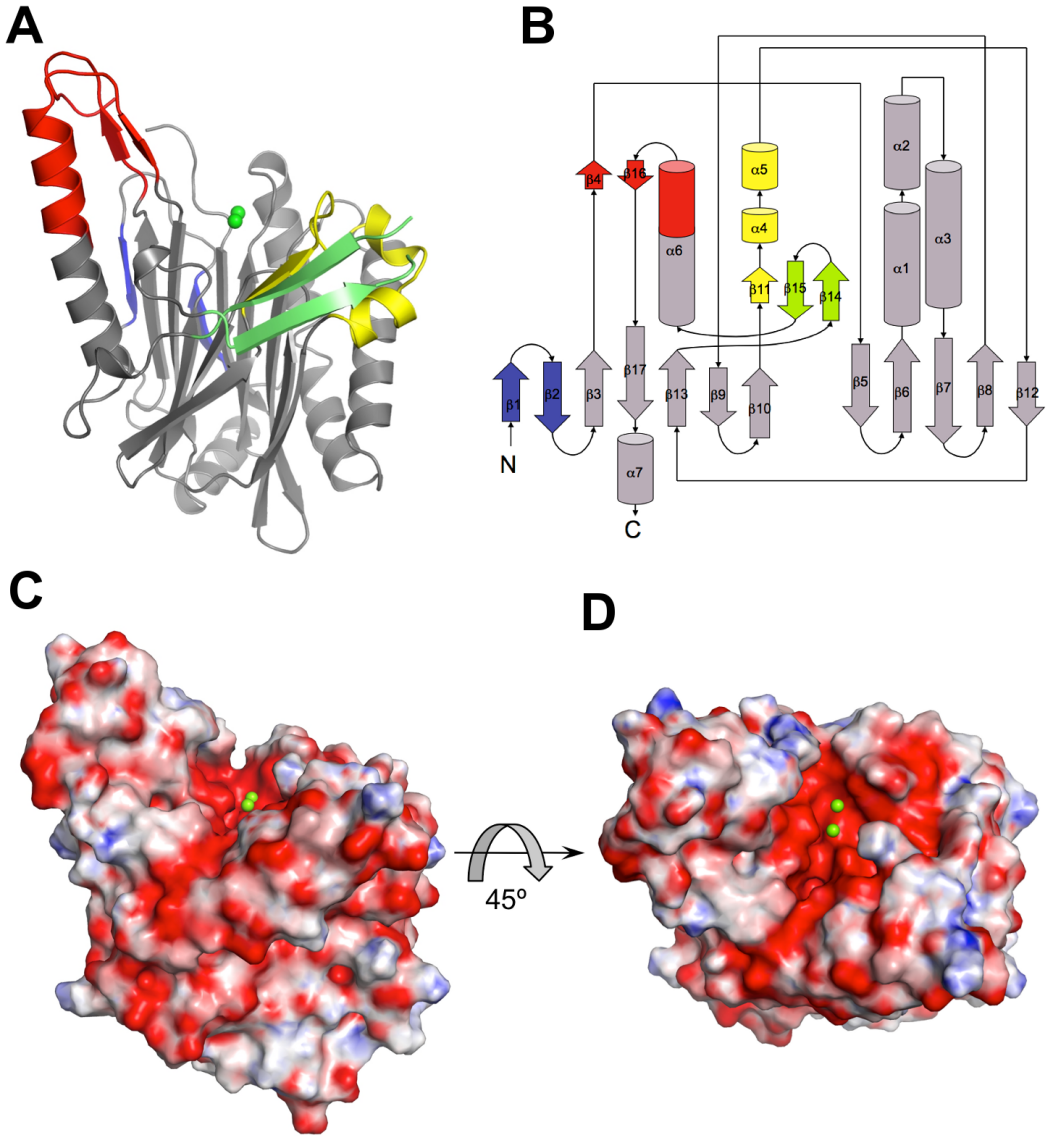
Crystal structure of the catalytic domain of SidD. (A) Ribbon backbone trace of SidD-NT (residues 37–350) highlighting the structural differences as compared to other PPM phosphatases. (B) topology diagram of SidD-NT colored as in (A): blue for the two extra β strands at the N-terminus, yellow for the flap domain, green for the β-sheet adjacent to the active site and red for the stalk-like protrusion. Helices are represented as cylinders and β strands as arrows. (C) Electrostatic potential surface of SidD-NT in the same orientation as in (A) Surfaces are colored based on an electrostatic potential gradient with positively charged regions in blue (+20 kcal per electron) and negatively charged regions in red (−20 kcal per electron). The two ions observed at the catalytic site are shown as green spheres. (D) Top view of the catalytic pocket colored accordingly to the same electrostatic potential gradient.

To further investigate the molecular basis for Rab1 recognition and de-AMPylation by SidD we initiated its structural characterization by X-ray crystallography. We identified a proteolytically resistant N-terminal domain (residues 37–350; SidD-NT) that fell within the domain borders of the largest catalytically active domain discovered above (Figure 1) and that crystallized readily. The structure of SidD-NT assumed an α/β fold formed by two stacked six-stranded antiparallel β-sheets flanked by α-helices (Figure 2A). Pairwise alignment using the DALI server [21] revealed a notable resemblance to metal-dependent protein phosphatases (PPMs), including human PP2Cα [22] and the bacterial PstP [23] which are considered the defining members of this family (Figure S2A). Despite the overall similarity to PPMs, SidD-NT exhibits several major structural differences (Figure 2A,B). First, SidD-NT contains two extra β strands at the N-terminus (β1 and β2) that contribute to extend the central β-sandwich as compared to the classical β-sandwich of the PstP bacterial phosphatase. A second difference resides within the region equivalent to the flap subdomain of PPMs. In most prokaryotic enzymes, the flap subdomain consists of a loop and two helical stretches connecting the last two strands of the central β-sandwich. The length and orientation of the flap region relative to the catalytic pocket is variable between different phosphatases and appears to regulate substrate binding and catalysis [24]. In the case of SidD-NT, the corresponding flap segment (residues 209–236) is completely repositioned by a large hinge bent tangential to the catalytic groove. Furthermore, the N-terminal section of the equivalent flap segment in SidD-NT contains a β strand (β11) that is part of a novel three-stranded antiparallel β-sheet adjacent to the active site. The other two strands (β14 and β15) of this extra β-sheet correspond to an insertion between α6 and β13. Finally, the third main structural difference corresponds to two additional insertions (residues 73–78 and 311–325) that contribute to a noticeable extension of helix α6 and the formation of a two-stranded β-sheet (β4 and β16). The extended α6 and the extra β-sheet form a stalk-like protrusion positioned on one side of the catalytic pocket. In summary, the crystallographic structure of SidD-NT assumes a PPM fold with some conformational rearrangements and the presence of additional subdomains, most of them being grouped around the negatively charged active site (Figure 2C, D).

### The active site

**Figure 3:**
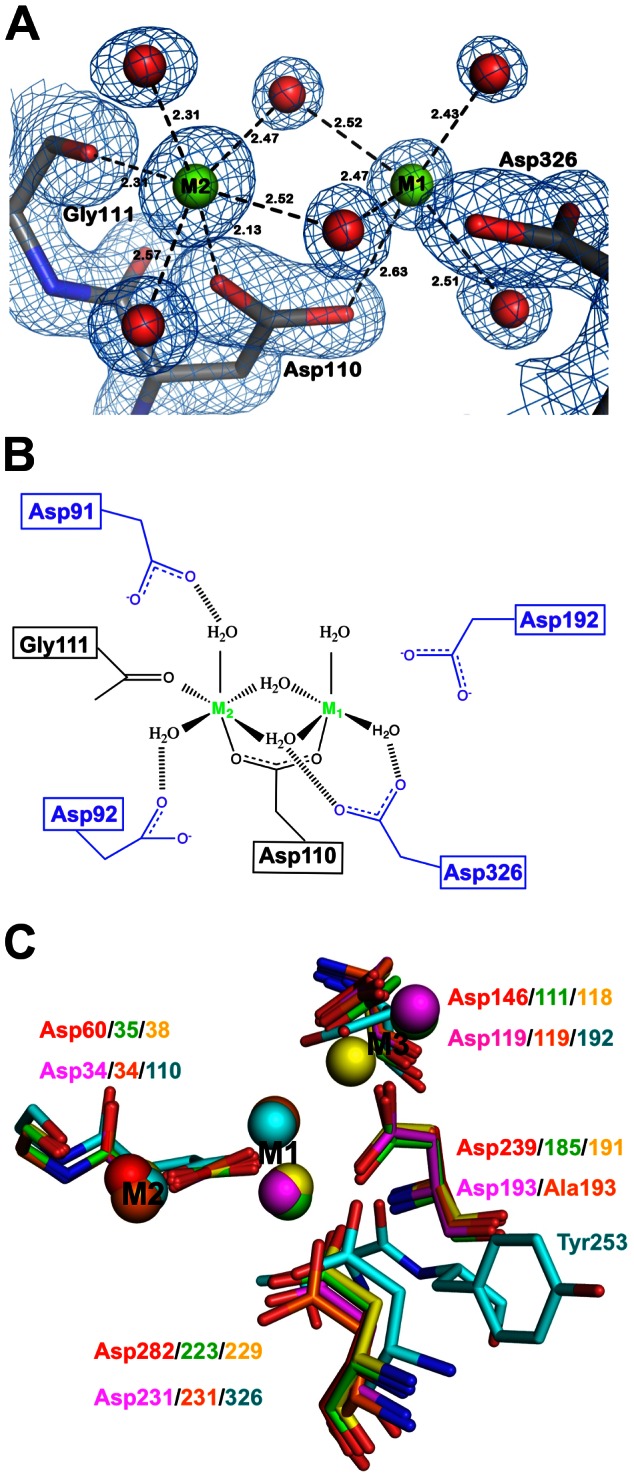
Structure of the catalytic site of SidD. (A) Difference electron density map (2F_o_-F_c_ contoured at 1.5σ, blue mesh) of the catalytic site of SidD showing the two-metal center and coordinating residues. Metals are shown as green spheres and water molecules as red spheres. (B) Schematic representation of the SidD active site shown in (A). (C) Superposition of the catalytic sites of PP2C (PDB 1A6Q) in red, MspP (PDB 2JFS) in green, PstP (PDB 1TXO) in yellow, tPphA (PDB 2J82) in pink, tPphA_D193A_ (PDB 2XVZ) in orange and SidD in cyan. Note the resemblance between the tPphA_D193A_ mutant and SidD. Both structures lack a highly conserved aspartic acid that coordinates simultaneously M1 and M3, and both structures exhibit a comparable M1 shifted position and the absence of M3 ion.

The active site of SidD-NT is located in a negatively charged cleft between the central β-sheets and comprises a relatively well-preserved binuclear metal center. The first metal (M1) is coordinated by four water molecules and residue D110, whereas the second metal (M2) is hexa-coordinated with the classical octahedral geometry formed by four water molecules, D110, and the main chain carbonyl of G111 (Figure 3A, B). The M1 position is slightly shifted as compared to other PPMs which can be attributed to the incomplete coordination derived from the absence of a highly conserved aspartic acid residue (Figure 3C). In this regard, D192 could accomplish the M1 hexa-coordination but the extended distance would require a conformational closure of the catalytic site. Interestingly, the conserved aspartic acid residue that is missing in the catalytic site of SidD coordinates a third ion (M3) in most bacterial PPMs (Figure 3C). The absence of this aspartic acid residue in SidD precludes a similar M3 coordination and no additional metal binding site is observed. Thus, in contrast to other bacterial PPM phosphatases, SidD appears to lack the capacity of binding a third ion at the equivalent M3 position.

Most PP2C phosphatases require either magnesium (Mg) or manganese (Mn) ions for their activity, with distinct preferences [25]. Quantitative Mg^2+^ analysis by inductively coupled plasma-optical emission spectrometry (ICP-OES) revealed a stoichiometry of Mg^2+^ relative to SidD of 1.7 to 1 (data not shown) suggesting that the active site of SidD contains two Mg^2+^ ions. Furthermore, mutation of D110A in SidD, which directly coordinates both M1 and M2 in the crystallographic structure, resulted in a dramatic reduction in the amount of Mg^2+^ to nearly negligible values. In this regard, the result from the quantitative ICP-OES analysis for Mg^2+^ correlates well with the two ions observed in the catalytic pocket of the SidD-NT structure.

**Figure 4:**
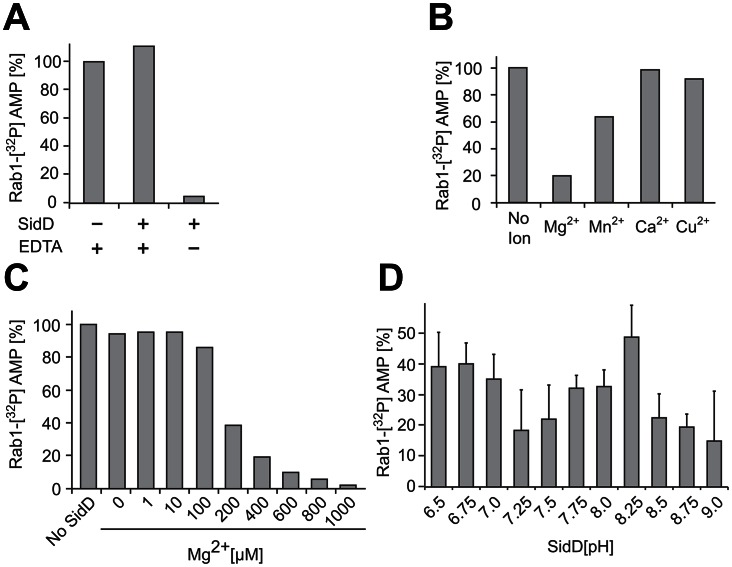
The de-AMPylation activity of SidD is Mg^2+^-dependent. De-AMPylation activity was measured by incubating GST-SidD with AMPylated Rab1 (2 µM) at a molar ratio of 1∶100. The amount of [^32^P]AMP-Rab1 remaining after 30 min (shown in %) was determined by scintillation counting. Each graph is a representative of two independent experiments with almost identical outcomes. (A) EDTA treatment interferes with de-AMPylation activity of SidD. Purified GST-SidD was washed with buffer with (+) or without (−) EDTA (20 mM) prior to incubation with Rab1-[^32^P]AMP. (B) Mg^2+^ ions restore the de-AMPylation activity of SidD. EDTA-treated GST-SidD was incubated with [^32^P]AMP-Rab1 in the presence of the indicated cation (600 nM final concentration). (C) The activity of SidD requires physiological levels of Mg^2+^. Same experiment as in (B) but with Mg^2+^ concentrations varying over a wide range. (D) The activity of SidD is pH-dependent as determined by de-AMPylation reactions executed in buffer with the indicated pH values.

The presence of Mg^2+^ ions within the catalytic pocket of SidD implied an important role of metal ions for the enzyme's activity. Consistent with this, we found that pre-incubation of SidD with the metal chelator ethylenediaminetetraacetic acid (EDTA) efficiently interfered with Rab1 de-AMPylation catalyzed by SidD (Figure 4A). Furthermore, the activity of SidD was fully restored by complementing the reaction with MgCl_2_ but not by adding other divalent ions such as calcium (Ca^2+^) or copper (Cu^2+^) (Figure 4B). A partial recovery of the de-AMPylation activity of EDTA-treated SidD was achieved when the reaction was supplemented with Mn ions (MnCl_2_). Together, these results indicate a strong preference of SidD for Mg^2+^ over other divalent ions which is further supported by the observation that SidD regained its maximum de-AMPylation activity at concentrations of 0.8–1.0 mM Mg^2+^ (Figure 4C) which correspond well with the physiological level of free Mg^2+^ [26].

Another notable difference between the catalytic site of SidD and other PPMs is the absence of a highly conserved arginine residue equivalent to R33 in PP2C, R17 in MspP, R20 in PstP and R13 in tPphA (Figure S2B), thought to play an important role for binding and neutralizing the negative charge of the phosphate monoester group during the catalysis [27]. The absence of this arginine side chain in SidD might reflect the difference in electrostatics between monoesterase and diesterase reactions, whereby the greater negative charge on the monoester (such as phospho-Tyr) relative to the diester phosphate (such as AMP-Tyr) might explain the necessity of an arginine side chain for stabilization. The pH dependency of PP2Cα in the presence of Mg^2+^ revealed the existence of two ionizable groups with pK_a_ values of 7.2 and 8.9 [28]. The lower pK_a_ has been interpreted as the binuclear bridging water which, in the form of a hydroxide ion, could attack the phosphorus substrate in a S_N_2-like mechanism. Using endpoint assays, we examined the pH-dependency of SidD's activity and observed two optimal pH values at ∼7.25 and ∼9.0 which suggest a ionization dependent catalytic mechanism (Figure 4D). Although the identity and protonation state of the amino acid side chains directly involved in the catalytic activity remains to be determined, the lower apparent pK_a_ value of SidD is comparable to that of PP2C [28], consistent with a similar binuclear bridging water acting as the reaction-initiating nucleophile. Indeed, D326 in the crystal structure of SidD, like D282 in PP2C, is appropriately positioned to accept the proton from the bridging water when the hydroxide ion is generated (Figure 3A). According to this interpretation, the de-AMPylation reaction performed by SidD involves a hydrolytic cleavage of the adenylyl-O-tyrosyl linkage, whereas the catalysis of the *E. coli* GS-ATase, the only other known de-AMPylase, utilizes a phosphorolysis mechanism in which a phosphate ion, not a hydroxyl ion, carries out the nucleophilic attack (Figure S3A, B).

### Ion-positioning residues are crucial for substrate hydrolysis by SidD

**Figure 5:**
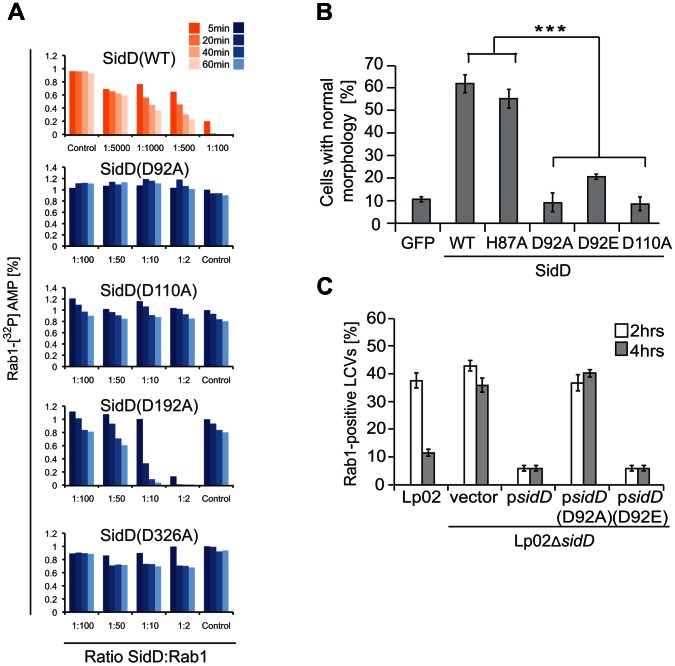
Mg^2+^ ion-positioning residues are crucial for SidD de-AMPylation activity. (A) In vitro de-AMPylation assay using purified recombinant proteins. SidD or the indicated SidD mutants were incubated with [^32^P]AMP-Rab1 (2 µM) at molar ratios listed underneath the graph. Note the different SidD-Rab1 ratios in graphs shown in blue vs orange. Samples were taken after 5, 20, 40, and 60 minutes (dark to light colors) and the amount of [^32^P]AMP-Rab1 remaining (shown in %) was determined by scintillation counting. (B) Cell rounding rescue assay. Transiently transfected COS1 cells simultaneously producing mCherry-SidM and either GFP or the indicated GFP-SidD mutants were analyzed by fluorescence microscopy for their cell morphology (for details see main text). The graph shows percentage of cells with normal morphology. Data are mean ± SD for three independent experiments. ***P<0.001 (two-tailed t test). (C) De-AMPylation-deficient SidD mutants are incapable of promoting Rab1 release from LCVs. Bone marrow-derived macrophages were challenged with either *L. pneumophila* wild type (Lp02) or with a *sidD* deletion strain (Lp02Δ*sidD*) complemented with either the empty vector or a plasmid producing the indicated SidD proteins (SidD, SidD[D92A] or SidD[D92E]). The infected cell monolayers were fixed at the indicated time points, and colocalization of host cell Rab1 with LCVs was determined by fluorescence microscopy. Data are mean ± SD for three independent experiments.

The structure of the active site revealed the presence of two Mg^2+^ ions coordinated by D92, D110, D326, the main chain carboxyl of G111, and several water molecules (Figure 3B). In addition, the nearby residue D192 could potentially fulfill the coordination of M1. In order to confirm the role of these residues in metal ion coordination, we created SidD mutant proteins in which each of the four aspartate residues was replaced with either alanine or with a similarly charged glutamate (Figure 5A; Figure S4). When assayed for [α^32^P]AMP removal *in vitro* we found that even a conservative substitution of aspartate for glutamate attenuated de-AMPylation activity of the SidD mutants considerably (D92E, D192E) or severely (D110E, D326E) (Figure S4). Upon a more drastic substitution of aspartate for alanine no residual activity was detected in three out of four SidD mutants (D92A, D110A, D326A) (Figure 5A). The recombinant mutant proteins displayed no detectable change in stability or solubility (Figure S4), suggesting the absence of major structural disturbances. In fact, we determined the crystallographic structure of SidD(D110A) at 1.9 Å resolution and confirmed the absence of coordinated Mg^2+^ ions within the catalytic pocket of the mutant protein without noticing any significant effect on its overall fold (Figure S3).

Next, we validated our *in vitro* de-AMPylation results in two independent mammalian cell-based assays. First, we analyzed the SidD point mutants for their ability to prevent SidM-induced COS1 cell rounding and cytotoxicity (Figure 5B). As expected, wild type GFP-SidD, which showed full de-AMPylation activity *in vitro*, prevented SidM-induced cytotoxicity in COS1 cells. In contrast, SidD(D92A) and SidD(D110A) were not capable of reducing the percentage of rounded cells that simultaneously produced SidM (Figure 5B), consistent with their lack of de-AMPylation activity *in vitro* (Figure 5A). SidD(D92E) which possessed residual de-AMPylation activity *in vitro* prevented morphological changes in twice as many COS1 cells as GFP alone (20% vs. 10%, respectively). Notably, the failure of SidD mutants to efficiently rescue cell rounding was not due to their inability to target to the Golgi compartment (Figure S4). In a second *in vivo* approach, we determined the effect of aspartate substitutions on the ability of SidD to catalyze de-AMPylation and, thus, removal of Rab1 from LCVs during the infection process (Figure 5C). Consistent with earlier reports [18], [19], *L. pneumophila* mutants lacking *sidD* showed a significantly prolonged colocalization with host cell Rab1 four hours post infection compared to LCVs containing wild type bacteria (36% vs 11% Rab1-positive vacuoles), in agreement with the failure of a *sidD* deletion strain to de-AMPylate Rab1 and to initiate Rab1 inactivation and removal from the LCV membrane by Rab1GAPs and GDI, respectively. The Rab1 removal defect of an *L. pneumophila* Δ*sidD* mutant was fully complemented by plasmid-encoded SidD but not by the catalytically inactive protein SidD(D92A). Remarkably, complementation with plasmid-encoded SidD(D92E) fully rescued the phenotype of a Δ*sidD* mutant, a phenomenon most likely attributable to the residual activity of this enzyme (Figure S4) which may have been further amplified by its overproduction from the high-copy plasmid within *L. pneumophila*. Taken together, our mutational analysis confirmed that the four aspartate residues at position 92, 110, 192, and 326 are crucial for SidD function both *in vivo* and *in vitro* (Figure 5) most likely by properly positioning the two catalytically essential Mg^2+^ ions inside the active site.

### The SidD-Rab1 binding interface

**Figure 6:**
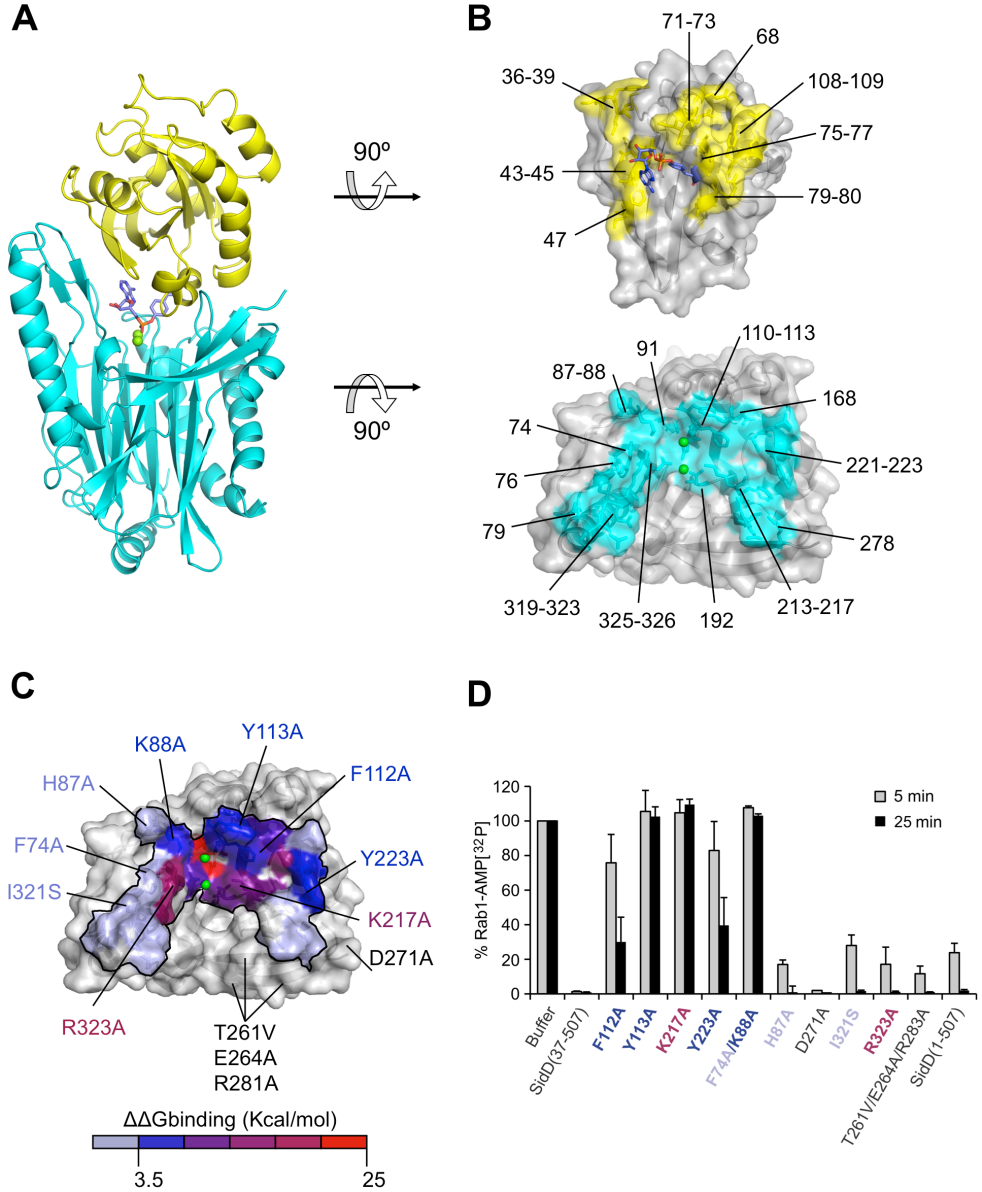
Structural model of the interaction between SidD-NT and Rab1. (A) Average structure of the complex between SidD (cyan) and AMPylated Rab1 (yellow) from the last nanosecond of the MD simulation. (B) Surface representation of Rab1 (top image) and SidD (bottom image) highlighting in yellow or cyan the residues that are within 4 Å distance to each other during the MD simulation. (C) View of the SidD interface surface contacting AMPylated Rab1, colored according to the computed effect of alanine mutations on the binding free energy (ΔΔ*G*). Note the hotspot in which the binding energy is largely concentrated. Arrows point to selected residues that were subsequently assessed in an *in vitro* de-AMPylation assay. (D) *In vitro* de-AMPylation assay using selected mutants located within and outside the binding hotspot shown in (C). [^32^P]AMP-Rab1 (2 µM) was incubated with SidD mutants at a molar ratio of 1∶100. The amount of [^32^P]AMP-Rab1 remaining after 5 and 25 min (shown in %) was determined by scintillation counting.

Despite significant efforts we were unsuccessful in obtaining crystals of the complex between SidD and either AMPylated and non-AMPylated Rab1. Furthermore, any attempts to crystallize SidD or the catalytically inactive mutant SidD(D92A) in the presence of AMP analogues such adenosine, adenosine 5′- monophosphate, 5′-(4-Fluorosulfonylbenzoyl)adenosine hydrochloride, adenosine 5′-(α,β-methylene)diphosphate, S-(5′-Adenosyl)-L-homocysteine, or 5′-Tosyladenosine were unsuccessful. Thus, to explore the interaction between both proteins we performed an energy-based rigid-body docking experiment with unmodified Rab1. By using the crystal structure of cacodylate bound to the MspP phosphatase [29] as initial constraint, we found that the docking solution with the Tyr77 hydroxyl O atom closest to the Mg ions (5.4 Å) was able to accommodate the AMP moiety in the same crystallized conformation without steric clashes. We then applied molecular dynamics (MD) in order to refine the SidD-Rab1(AMP) docking model as well as to evaluate its stability. In this regard, the initial docking showed only small fluctuations along the MD simulation indicating a stable SidD-Rab1 interaction (Figure S5A). Similarly, the Mg^2+^-phosphate interaction at the catalytic site remained constant during the MD simulation (Figure S5B). These results further attested a good structural complementarity between SidD and AMPylated Rab1 with a buried surface of approximately 1,300 Å^2^ and unrestricted access to the catalytic pocket without the need of large conformational rearrangements (Figure 6A,B). Next, we used *in silico* alanine scanning on the interfacial residues of SidD to predict relevant hotspots for Rab1 recognition. Interestingly, the residues with higher contribution to the binding free energy are grouped asymmetrically around the catalytic pocket (Figure 6C, and Table S2 in Text S1). Indeed, the average structure from the last nanosecond of the MD shows that F112_SidD_ and Y113_SidD_ form extensive hydrophobic interactions with Y77_Rab1_ (Figure S5C). Another participating residue is K217_SidD_ which is facing the phosphate group of AMP and may function as proton donor for the leaving phosphate. More peripherally, Y223_SidD_ contributes to the hydrophobic burial of Y109_Rab1_. Other residues such as E168_SidD_ and D221_SidD_ form hydrogen bonds with R79_Rab1_ (Figure S5C). Finally, the docking model shows that the AMP moiety is accommodated in a groove adjacent to the catalytic pocket of SidD without being detached from Rab1 (Figure 6A). The adenine base of AMP rests against F74_SidD_ and K88_SidD_ and lacks additional specific interactions whereas the ribose hydroxyl groups interact with R323_SidD_.

To validate the binding hotspot found in SidD, we mutated several residues that contribute to the interaction with Rab1 and examined the effect on the ability of SidD to remove [α^32^P]AMP from Rab1 *in vitro*. In agreement with the interactions described above, the single residue substitutions F112A, Y113A, K217A, Y223A, and Y253E as well as the double exchange F74A/K88A strongly affected the ability of SidD to de-AMPylate Rab1 (Figure 6D) without compromising the overall protein stability or solubility (Figure S5). Only the R323A mutant, designed to disrupt the interaction with the ribose of AMP, had no apparent effect on SidD activity, which may reflect a redundant interaction as a consequence of nearby contacts. Notably, while replacement of Y113 with glutamine strongly reduced Rab1 de-AMPylation by SidD, substitution with the structurally similar phenylalanine had no apparent effect on activity (Figure S4), consistent with phenylalanine but not glutamine being capable of mediating π stacking interactions with Y77_Rab1_. We also examined additional mutations outside the binding hotspot such as I321S, D271A, or H87A and even the triple mutant T261A/E264A/R281A and, as expected, observed no obvious reduction in SidD activity *in vitro* (Figure 6C, D) or *in vivo* (H87A; Figure 5B) which further validated the SidD-Rab1 docking model. Overall, the experimental results are in remarkable agreement with the model complex, with the majority of the mutations at the binding hotspot severely attenuating or preventing SidD-mediated de-AMPylation of Rab1.

### SidD catalysis is specific for Rab1 and AMP

**Figure 7:**
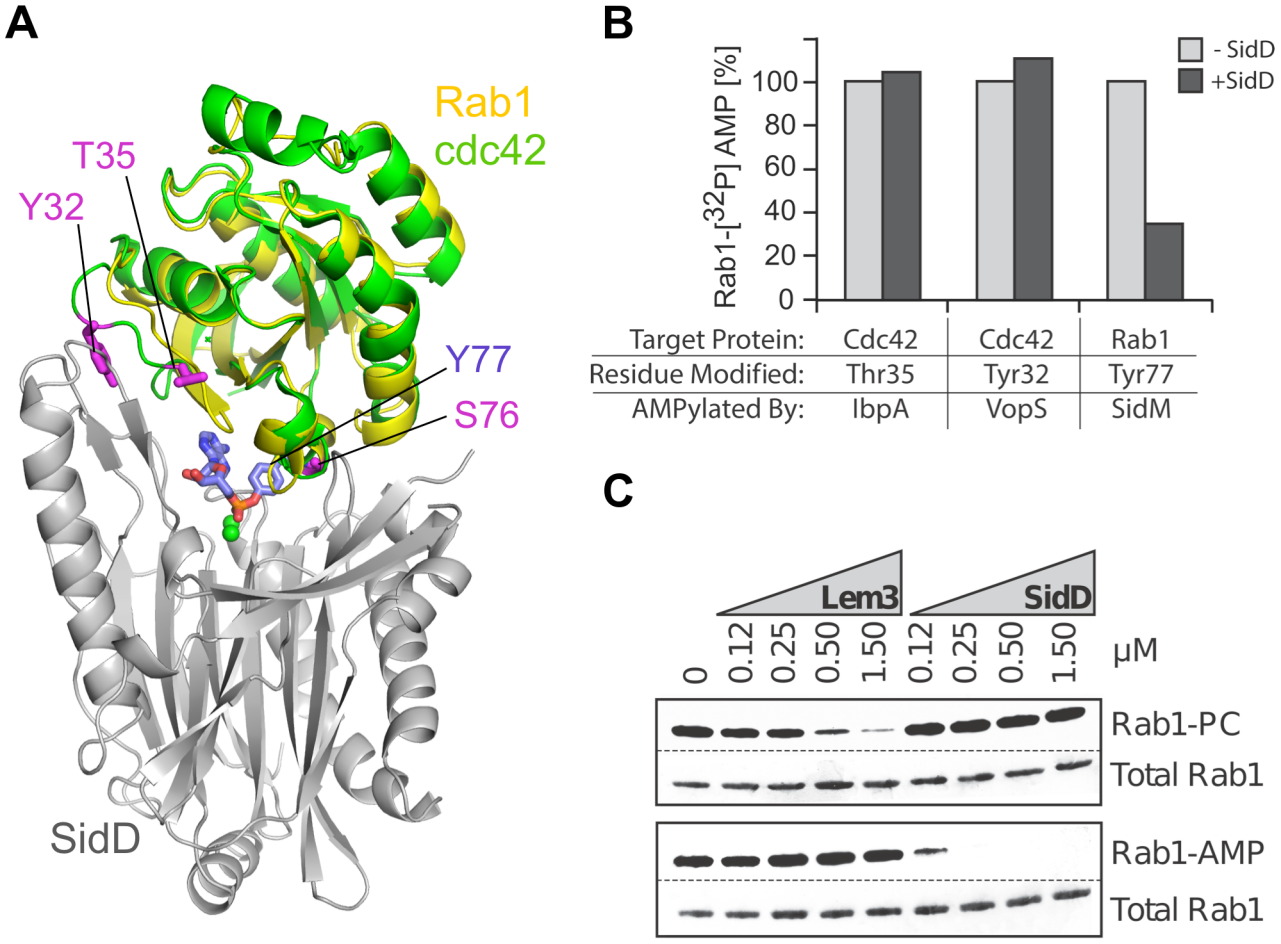
SidD de-AMPylates Rab1 but not Rho GTPases. (A) Superposition of Rab1 (yellow) and Cdc42 (green) demonstrating the location of Y32_Cdc42_ and T35_Cdc42_ relative to Y77_Rab1_. (B) In vitro de-AMPylation assay using purified recombinant proteins. Cdc42 or Rab1 were exposed to either IbpA, VopS, or SidM (as indicated) to covalently modify the GTPases at the indicated amino acid residues followed by incubation with SidD at a molar ratio of 1∶100. The amount of [^32^P]-labeled Cdc42 and Rab1 remaining after 5 minutes was determined by scintillation counting. (C) Lem3 and SidD exhibit substrate specificity for Rab1-PC or Rab1-AMP, respectively. Purified phosphocholinated Rab1 (10 µM) or AMPylated Rab1 (10 µM) were incubated with buffer or with increasing amounts of either Lem3 or SidD as specified in the figure for 2 hours at room temperature. Total Rab1 was determined by Ponceau staining of the proteins transferred from an SDS-PAGE gel to a nitrocellulose membrane, while Rab1-PC and Rab1-AMP were detected by Western blot using anti-phosphocholine or anti-AMP antibodies, respectively.

The structure of SidD displays a stalk like protrusion on one side of the active site cleft and a binding hotspot on the other side, features that may contribute to recognizing and properly orienting Rab1 in a way that the AMPylated Y77_Rab1_ is correctly positioned inside the catalytic pocket. We speculated that this topological design might allow SidD to distinguish AMPylated Rab1 from similarly modified substrates such as Rho GTPases. To validate our hypothesis, we generated [^32^P]AMP-labeled Cdc42 by incubating it with either *V. parahaemolyticus* VopS, which AMPylates Cdc42 at Y32, or with *H. somni* IbpA, which AMPylates the neighboring T35 (Figure 7A), and tested the ability of SidD to convert Cdc42 back into the unmodified form. In contrast to [^32^P]AMP-Y77_Rab1_ which was efficiently de-AMPylated by SidD, neither [^32^P]AMP-T35_Cdc42_ nor [^32^P]AMP-Y32_Cdc42_ showed any detectable decrease in the AMPylation level in the presence or absence of SidD (Figure 7B). Thus, SidD did not accept Cdc42 as substrate for de-AMPylation *in vitro* even if the AMP modification in Cdc42 was located on a tyrosine residue (Y32), as it is the case in AMPylated Rab1 (Y77). Similar results were obtained in an *in vivo* assay where SidD failed to prevent rounding of COS1 cells transiently producing GFP-tagged VopS (Figure S6) confirming that Rho GTPases AMPylated by VopS cannot be de-AMPylated by SidD.

Finally, we determined if SidD could remove posttranslational modifications other than AMP from Rab1. The *L. pneumophila* effector AnkX/LegA8 covalently attaches phosphocholine to serine-76 in Rab1 (S76_Rab1_), the residue located immediately adjacent to Y77_Rab1_, the target of AMPylation by SidM [30]. Like AMP, phosphocholine is connected to Rab1 via a phosphodiester bond. Its removal requires the *L. pneumophila* effector Lem3 [31], [32], [33] which we predict assumes a PP2C-like fold similar to SidD (data not shown). Given the similarity of the two removing enzymes and of the chemical bond they hydrolyze we explored whether SidD or Lem3 are capable of catalyzing the other enzyme's reaction. While AMP was efficiently removed by SidD and phosphocholine by Lem3, neither modification was affected by the presence of the opposite enzyme (Figure 7C). Together, these data favor the idea that SidD from *L. pneumophila* (and probably Lem3 as well) has evolved to exclusively recognize its host cell substrate and to remove only a particular post-translational modification from a specific side chain location.

## Discussion

To our knowledge, SidD from *L. pneumophila* is the first known microbial effector protein with de-AMPylation activity. Together with the AMPylase SidM it forms an enzymatic cascade that enables the pathogen to post-translationally modify host cell Rab1 in a transient rather than permanent manner. Despite limited sequence homology, the crystal structure of the de-AMPylation domain of SidD revealed a notable similarity to Serine/Threonine phosphatases of the PPM family. However, in addition to the conserved PPM core, SidD-NT exhibits additional structural elements like a repositioned flap domain as part of a new three-stranded antiparallel β-sheet and a stalk-like protrusion, both derived from sequence insertions located around the catalytic site, thus with potential regulatory functions (Figure 2A, B). The finding that SidD is a PPM phosphatase with de-AMPylation activity constitutes a clear example of how *L. pneumophila* has adapted a common enzymatic fold and mechanism to effectively hydrolyze an unusual substrate. In contrast, AT-N from the *E. coli* GS-ATase assumes a nucleotidyl transferase fold, indicating that de-AMPylases have developed more than once during microbial evolution. From a chemical perspective, a common feature of enzymes that hydrolyze phosphate monoesters and diesters is the presence of a binuclear metal center. PPM phosphatases share an invariant M1 and M2 whereas the presence of an additional M3 in bacterial homologs is associated with a small flap subdomain adjacent to the catalytic site. The role of this M3 is still unclear, although it has been proposed to modulate the flap orientation and, thus, substrate binding [34]. More recently, the M3 has been associated with the activation of a water molecule that might function as a proton donor for the leaving phosphate [35]. The crystal structure of SidD-NT shows the absence of an absolutely conserved aspartate in the catalytic site that in other PPMs coordinates M1 and M3. This absence not only produces a slight shift in the M1 position but also compromises the coordination of a third ion. Indeed, the quantitative ICP-OES analysis of SidD together with the ion-dependent enzymatic activity assay (Figure 4) support the presence of two Mg^2+^ ions that are essential for Rab1 de-AMPylation. Collectively, the absence of an M3, the M1 shifted position, the strict requirement of Mg^2+^ ions for catalysis, and the absence of an arginine side chain to interact with the phosphate group appear to be variations through which *L. pneumophila* SidD has been converted into an enzyme that de-AMPylates Rab1, capitalizing on the existing PPM active site.

The crystal structure of cacodylate bound to the MspP phosphatase shows a direct interaction with M1 and M2 by bidentate coordination which has been interpreted as a mimicking phospho-substrate intermediate during the catalysis [29]. By using this metal-phosphate coordination as initial constraint in docking AMPylated Rab1 into the catalytic pocket of SidD, we found a remarkable surface complementarity (Figure 6). Subsequent analysis of the docking model by molecular dynamic simulations showed that both the root mean square deviation (RMSD) of the complex with respect to the initial model as well as the distance between the two Mg^2+^ ions and the phosphate group of AMP experienced only small fluctuations during the simulation process (Figure S5). These observations not only confirm the stability of the docking prediction but, more importantly, evidence a good structural complementarity between SidD and AMPylated Rab1 without the need for large conformational rearrangements to access the catalytic pocket. It should be noted that although our docking model is energetically favorable, the AMP-Tyr side-chain could adopt alternative conformations relative to the crystallized AMPylated Rab1 and that the actual protein complex may experience additional structural rearrangements beyond what has been sampled in our simulations. We also analyzed the interface features of SidD that enable the initial recognition of AMPylated Rab1. Using computational alanine scanning, we identified a hotspot in which the binding energy is largely concentrated on a few amino acids near the catalytic pocket. Indeed, the majority of individual mutations introduced at the binding hotspot severely attenuated or prevented the catalytic activity of SidD *in vitro* (Figure 6D), which is in remarkable agreement with the qualitative description of the SidD-Rab1 interaction derived from our docking model.

Our structural, computational and mutational analysis revealed the existence of distinctive features in SidD such as the binding hotspot flanking the catalytic site or the stalk-like protrusion that appear to be absent from generic phosphatases. We hypothesized that through this topological design SidD can distinguish AMPylated Rab1 from similarly modified substrates. Accordingly, we demonstrated that AMPylated Rho GTPases were not recognized by SidD under any of the conditions where Rab1 was efficiently de-AMPylated (Figure 7B, Figure S6). Likewise, we found that phosphocholinated Rab1 did not serve as substrate for SidD (Figure 7C) even though its post-translational modification was comparable to AMPylated Tyr77_Rab1_ with respect to its location (Ser76_Rab1_) and chemical linkage (phosphodiester bond). The fact that the activity of the de-phosphocholinase Lem3 was similarly restricted from targeting AMPylated Rab1 (Figure 7) suggests that these *L. pneumophila* effectors have adapted their catalytic activity towards their correct host target thought the acquisition of specific topological determinants.

Based on our domain mapping and cellular localization studies (Figure 1) we predict the existence of a second functional region in SidD that assists in localizing the protein to membranes, more precisely the LCV membrane within *L. pneumophila*-infected cells or the Golgi compartment within transiently transfected cells. The exact mechanism of membrane targeting of SidD, however, remains unclear. Several *L. pneumophila* effectors have been shown to specifically interact with phospholipids such as PI(3)P or PI(4)P in order to associate either with the LCV membrane or with other host cell compartments [36]. Indeed, targeting of effectors to a specific cellular compartment constitutes an additional mechanism to enhance substrate specificity. Using protein-lipid overlay assays we were unable to detect binding of SidD to any of the most common phosphoinositides (data not shown), suggesting that membrane targeting of the C-terminal region is mediated by binding to another lipid or proteinaceous host factor. Several attempts to demonstrate a stable association between purified recombinant SidD and Rab1 by pulldown studies failed, indicating that the interaction between both proteins is of transient nature. Nonetheless, it is likely that even a weak interaction with Rab1 is sufficient to retain the majority of SidD molecules in close proximity to the LCV membrane after their translocation by the *L. pneumophila* T4SS. It is worth mentioning that the prenylation anchor of Rab1 and the C-terminal targeting region of SidD are located on the same side of the complex thus with the potential to simultaneously contact the LCV membrane during catalysis, further strengthening the likelihood of our modeled complex (Figure S7). Future studies should help to reveal the mechanistic details of SidD membrane targeting and substrate detection during host cell infection.

In summary, the study presented here provides an important first look at the structure and catalytic mechanism of SidD and reveals that this *L. pneumophila* effector differs in many aspects from the *E. coli* GS-ATase, the only other known de-AMPylase. The finding that SidD is a converted phosphatase equipped with structural elements designed to distinguish AMPylated Rab1 from similar host cell substrates demonstrates the versatility of the phosphatase fold and suggests that it may have served as blueprint for a variety of thus far uncharacterized de-modifying enzymes capable of targeting an array of different post-translational modifications.

## Materials and Methods

### Crystallization and structure determination

Native SidD_37–350_ was concentrated to 8 mg/ml and used for initial crystal screening. All crystallization conditions were carried out in a sitting drop setup of 0.1 µL protein solution mixed with 0.1 µL of mother liquor. Visible crystals appeared in several comparable conditions after 5 days at 18°C. Further optimization yielded good quality crystals in 1.6–1.8 M NaCl, 0.1 M NaOAc pH 4.8, 20% glycerol using 2 µL sitting drops with equal protein/mother-liquor ratio. The D110A mutant crystallized under the same conditions as the native SidD_37–350_.

The structure of SidD_37–350_ was solved by single anomalous dispersion with isomorphous replacement (SIRAS) using a single gadolinium derivative [37]. Gd positions were determined using the SHELX software [38]. The initial electron density map was then calculated with experimental phases derived from the Gd positions with phenix.phaser [39]. A preliminary model was automatically traced by phenix.autobuild and completed by hand in Coot [40]. The model was improved through alternating cycles of manual rebuilding using Coot and refinement using phenix.refine. A final refinement cycle was performed with REFMAC5 [41], [42]. This model was subsequently used for molecular replacement with the high-resolution diffraction data using phenix.phaser. Additional model building and refinement was performed using Coot, phenix.refine and REFMAC5. The final models have good structural geometries with no residues in disallowed regions of the Ramachandran plot. Statistics on data collection and refinement are provided in Table S1 in Text S1. All the molecular representations were prepared with PyMOL (The PyMOL Molecular Graphics System, Schrödinger, LLC) and ChemDraw (PerkinElmer).

### Structural model of the Rab1/SidD complex by docking

We built a structural model of Rab1/SidD complex by rigid-body docking, based on the FFT-based docking program Zdock2.1 [43] and the energy-based pyDock scoring scheme [44]. Details of this procedure are described in Supplemental Materials and Methods.

### Refinement of the docking model by molecular dynamics

In order to refine the docking model of the Rab1(AMP)/SidD complex we performed molecular dynamics (MD) simulation in explicit solvent using the force field AMBER parm99 of the AMBER10 package [45], [46]. Details of this procedure are described in Supplemental Materials and Methods.

### Identification of key residues for the interaction between SidD and AMPylated Rab1

We performed *in silico* alanine scanning on the MD refinement of the selected docking model to identify the key residues responsible for the binding process. The *MMPBSA.py* script in AMBER12 [47] was used to carry out all binding energy calculation using the MM-GBSA method on 200 snapshots extracted from the last 2 ns of the MD trajectory of the selected docking model. Each SidD interface residue was mutated to alanine and then we estimated the binding free energy change (ΔΔG) as the difference between the binding ΔG of the wild type and the mutated complex (van der Waals and electrostatic energy by the MM force field, electrostatic contribution to the solvation free energy by GB method, and nonpolar contribution to the solvation free energy by an empirical model). The conformational entropy contribution to binding was not included here, given the difficulty of computing it for a large protein-protein complex, and the small effect when calculating relative system free energies.

### AMPylation and de-AMPylation assays

#### Radioactive assays

[^32^P]AMP-labeled Rab1 was generated as described [3], [18]. Briefly, Rab1 (7.5 µM) was incubated with 7×10^7^ SidM-coated Dynabeads magnetic beads M-270 Epoxy (LifeTechnologies) and incubated for 4 hours at room temperature in PBS buffer supplemented with 25 nM [α^32^P]ATP, 7.5 µM cold GTP, 1 mM MgCl_2_, and 1 mM β-ME. Cdc42 was AMPylated for 2 hours at room temeprature through incubation with a 1 to 100 molar ratio of VopS to Rab1 in a buffer containing 20 mM HEPES pH 7.4, 5 mM MgCl_2_, and 100 mM NaCl or in the presence of a 1 to 5 molar ratio of IbpA Fic1 to Rab1 in PBS buffer supplemented with 1 mM MgCl_2_ and 1 mM β-ME (PBS-MM). A 5 x molar access of non-radiolabeled ATP was added and the reaction was incubated for 1 hour at room temperature to fully AMPylate Cdc42.

De-AMPylation of 2 µM Rab1a-[^32^P]AMP was initiated by addition of 40 nM purified His-SidD point mutants or wild type. Alternatively, de-AMPylation of 2 µM Rab1-[^32^P]AMP was initiated by GST-SidD full length, fragments, and point mutants at the indicated molar ratios in PBS with 4 µM GTP, 10 µM non-radiolabeled ATP. The initial reaction volume was 100 µl and at each time point a 20 µl sample was removed from the reaction and loss of [^32^P]AMP from Rab1a was monitored by nitrocelluloase filter-binding assays as previously described [18].

To determine ion dependency, all reaction components (Rab1a, SidM, and SidD) were dialyzed against ion-free PBS in the presence of 10 mM EDTA. EDTA-treated SidD was then dialyzed against either ion-free PBS or PBS supplemented with the indicated cations and tested for de-AMPylation activity as described above.

To determine pH dependency of SidD's de-AMPylation activity, Rab1A (25 µM) was first AMPylated in PBS in the presence of 50 nM [α^32^P]ATP and 1.2×10^9^ Dynabeads (Invitrogen) coated with SidM in a total reaction volume of 1.2 ml. After 4 hours at room temperature, Rab1A-AMP[^32^P] was separated from the SidM-coated Dynabeads and 100 µL aliquots were subjected to buffer exchange against the constant ionic strength buffer 0.1 M ACES, 52 mM Ethanolamine, and 52 mM Tris (ACES-ET) with pH ranging from 6.5 to 9 [48]. The buffer exchange was performed using Zeba Spin Desalting Columns (Pierce) according to manufacturer instructions. Rab1A-AMP[^32^P] (6.25 µM) was de-AMPylated in the presence of 1 mM MgCl_2_ and 62.5 nM GST-SidD at room temperature and after 5 minutes the reaction was stopped by addition of ACES-ET pH 5.5.

#### Non-radioactive assays

Rab1a was AMPylated and purified by gel filtration as previously described [18].

For de-AMPylation, Rab1-AMP (10 µM) was incubated for 2 h at room temperature with increasing amounts of the purified SidD or Lem3 in PBS-MM. The protein samples (∼2 µg total Rab1a) were then separated on a 4–15% SDS–PAGE gel (BioRad) and transferred to a nitrocellulose membrane (iBlot, Invitrogen) for immunoblot analysis (Fast Western, Pierce) using anti-AMP rabbit polyclonal antibody (BellBrook Labs) to detect AMPylated Rab1a.

### Phosphocholination and de-phosphocholination assays

Rab1a (25 µM) was phosphocholinated at room temperature for 4 h in the presence of His-AnkX (0.25 µM) in a buffer containing 20 mM HEPES pH 7.5, 100 mM NaCl, 1 mM CDP-choline, 1 mM MgCl_2_, and 1 mM ATP. The reaction mixture was then incubated with 60 µl of HisLink beads (Promega) to remove His-AnkX before purification by gel filtration on a HiLoad 16/60 Superdex 75 pg column (GE Healthcare) at 4°C. Fractions containing phosphocholinated Rab1a (Rab1-PC) in either PBS-MM were pooled, concentrated, and stored at −80°C.

For de-phosphocholination, Rab1-PC (10 µM) was incubated for 2 h at room temperature with increasing amounts of the purified Lem3 or SidD in PBS-MM. Immunoblot analysis was used as described above for the de-AMPylation assays using the anti-phosphocholine-specific antibody TEPC-15 (Sigma) to detect phosphocholinated Rab1a.

### Immunofluorescence microscopy

Immunofluorescence microscopy was performed as previously described [18].

### Accession numbers

The structural coordinates of SidD_37–350_ and SidD_37–350_(D110A) have been deposited in the Protein Data Bank (http://www.rcsb.org.pdb) with the accession codes 4IIK and 4IIP respectively.

## Supporting Information

Figure S1 **GFP-SidD variants are stably produced in COS1 cells.** Transiently transfected COS1 cells producing GFP-SidD fragments were harvested, resuspended in sample buffer, and proteins were separated by SDS-PAGE followed by Western blot analysis using anti-GFP antibody.
(TIF)

Figure S2 **Comparison of SidD with other PPM phosphatases.** (A) Superposition of PP2C (phosphatase 2C, PDB 1A6Q) in red, MspP (PPM phosphatase from *Mycobacterium smegmatis*, PDB 2JFS) in yellow, PstP (PPM phosphatase from *Mycobacterium tuberculosis*, PDB 1TXO) in green, tPphA (PPM phosphatase from *Thermosynechococcus elengatus*, PDB 2J82) in pink and SidD in cyan. Domains with no structural homology outside the phosphatase core have been omitted for clarity. (B) Structure-based sequence alignment between the same PPM phosphatases descrived in (A) and SidD. Strictly conserved residues are highlighted in red. Conserved arginine residues, considered to be important for binding the phosphate monoester group during the catalysis are highlighted in yellow. Structural differences present in SidD are colored on the upper diagram as in Fig. 2 A,B.
(TIF)

Figure S3 **The catalytic center of SidD.** (A) Schematic presentation of the mechanism proposed for AT-N based on the reverse reaction catalyzed by some nucleotidyl transferases such as DNA polymerase β and poly(A) polymerase. (B) Schematic view illustrating the catalytic mechanism of SidD adapted from the general mechanism proposed for the PPM phosphatase MspP. (C) Superposition of the structures of wild-type SidD in cyan vs D110A mutant in pink color. (D) Difference electron density map (2F_o_-F_c_ contoured at 1.5σ, blue mesh) of the catalytic site of the wild-type SidD in stereoview representation (upper panel) and the same view of the SidD(D110A) mutant showing the absence of coordinated ions (lower panel).
(TIF)

Figure S4 **Functional analysis of SidD mutants.** Continuation of the de-AMPylation experiment shown in Fig. 5A showing the *in vitro* de-AMPylation activity of SidD mutants. (A) Analysis of mutants in which aspartate residues at position 92, 110, 192, or 326 have been replaced with similarly charged glutamate. (B) SidD mutants where Y113 has been substituted with either glutamine or the structurally similar phenylalanine. (C) Analysis of SidD mutant protein stability and intracellular localization. Mutant proteins with N-terminal GST-tag purified from *E. coli* were analyzed by SDS-PAGE and Coomassie staining. (D) Localization of GFP-tagged SidD(D92A) within transiently transfected COS1 cells. SidD mutant proteins showed intracellular localization reminiscent of the wild type protein (Fig. 1C main text), suggesting that their failure to rescue SidM-induced rounding of COS1 cell was not due to a defect in proper targeting of the protein.
(TIF)

Figure S5 **Analysis of the SidD-Rab1 interface.** (A) Plot of root mean square deviation (RMSD) of SidD in complex with AMPylated Rab1. (B) Plot of the distances between the non-bridging phosphoryl oxygens of the AMP and the Mg^2+^(1) and Mg^2+^(2) catalytic ions of SidD. Note the stable distances over the simulation period. (C) Close-up view of the binding interface residues between SidD and Rab1 from the average structure from the last nanosecond of the MD simulation. (D) Mutant proteins with N-terminal GST-tag purified from *E.coli* were analyzed by SDS-PAGE and Coomassie staining for their stability and solubility. *: SidD(37-507) variants.
(TIF)

Figure S6 **Functional specificity of SidD.** (A) VopS-induced cytotoxicity is not rescued by SidD. COS1 cells co-transfected with plasmids encoding mCherry-VopS and GFP-SidD were fixed after 12 hours, nuclei were labeled using Hoechst stain, and nuclear morphology of doubly-transfected and untransfected (*) cells was determined by fluorescence microscopy. Scale bar, 1 µm. (B) Quantification of the experiment shown in (A) showing the percentage of cells with regular nuclear morphology. COS1 cells overproducing plasmid-encoded mCherry-VopS showed extensive cell rounding and nuclear condensation which was not observed in control cells producing mCherry (>90% vs <15%, respectively). The simultaneous presence of either GFP-SidD or GFP alone did not noticeably reduce VopS-induced nuclear condensation, further confirming that Cdc42 was not a substrate for SidD-catalyzed de-AMPylation. Data were obtained from two independent experiments.
(TIF)

Figure S7 **Structural model for the specific recognition of AMPylated Rab1 by SidD.** Rab1 is anchored to the LCV membrane through its C-terminal hydrophobic prenyl tails whereas SidD is targeted to the same membrane via its C-terminal domain. Then through complementary shape, charge and hydrophobic interactions the N-terminal domain of SidD binds to AMPylated Rab1 and catalyzes the hydrolysis of the phosphodiester bond between AMP and Tyr77. The configuration of the complex shows how the prenylation anchor of Rab1 and the C-terminal targeting region of SidD are oriented towards the LCV membrane. Yellow, Rab1 in ribbon backbone representation with transparent surface; cyan, SidD in ribbon backbone representation with transparent surface; violet, Tyr77-AMP in stick representation; green spheres, Mg^2+^ ions; Lilac, prenyl groups.
(TIF)

Text S1 **Additional details of methods used for protein production, purification, X-ray data collection, quantitative elemental analysts, SidD-Rab1 model docking, and refinement by molecular dynamics.**
(DOCX)
