# Correction: Structural Basis for Rab1 De-AMPylation by the *Legionella pneumophila* Effector SidD

**DOI:** 10.1371/annotation/2b8e6418-2496-4ffb-b230-95d90b748086

**Published:** 2013-07-30

**Authors:** Yang Chen, Igor Tascón, M. Ramona Neunuebel, Chiara Pallara, Jacqueline Brady, Lisa N. Kinch, Juan Fernández-Recio, Adriana L. Rojas, Matthias P. Machner, Aitor Hierro

Two errors were made in this article. First, a reference was omitted from the second sentence of the fourth paragraph in the Introduction section. 

The sentence should read: SidD lacks any obvious sequence homology with the AT-N or other known proteins, although fold recognition analysis of the N-terminal portion of SidD predicted limited resemblance with members of the metal-dependent protein phosphatase (PPM) family (Rigden 2011).

The reference is: Rigden DJ. (2011) Identification and modelling of a PPM protein phosphatase fold in the Legionella pneumophila deAMPylase SidD. FEBS Lett. 585:2749-54.

doi: 10.1016/j.febslet.2011.08.006.

Second, there were some omissions from the acknowledgements section, which should instead read as follows:

We thank K. B. Decker, Y. Lin, and A.H. Gaspar for comments on the manuscript, and A. Vidaurrazaga for assistance with biochemical experiments. This study made use of the European Synchrotron Radiation Facility (ESRF, Grenoble, France) under the Block Allocation Group (BAG) MX1420, the Diamond Light Source (Oxfordshire, UK) under the rapid access mode MX7512 and BAG MX8302, the beamline PROXIMA1 at the SOLEIL synchrotron (Saint-Aubin, France), the X-ray crystallography platform and proteomics platform (member of CIBERehd and ProteoRed-ISCIII) at CIC bioGUNE (Derio, Spain), and the SGIker analytical facility at UPV/EHU (Leioa, Spain). We thank all the staff from these facilities for technical and human support. Access to synchrotron facilities was part funded by the the BioStruct-X project (proposal number 2460).

