# Supplementary figures and images for: Structural Basis for Rab1 De-AMPylation by the Legionella pneumophila Effector SidD
Source: PLoS Pathog. 2013 May 16;9(5):e1003382. doi: 10.1371/journal.ppat.1003382 (PMC3656104; doi:10.1371/journal.ppat.1003382)

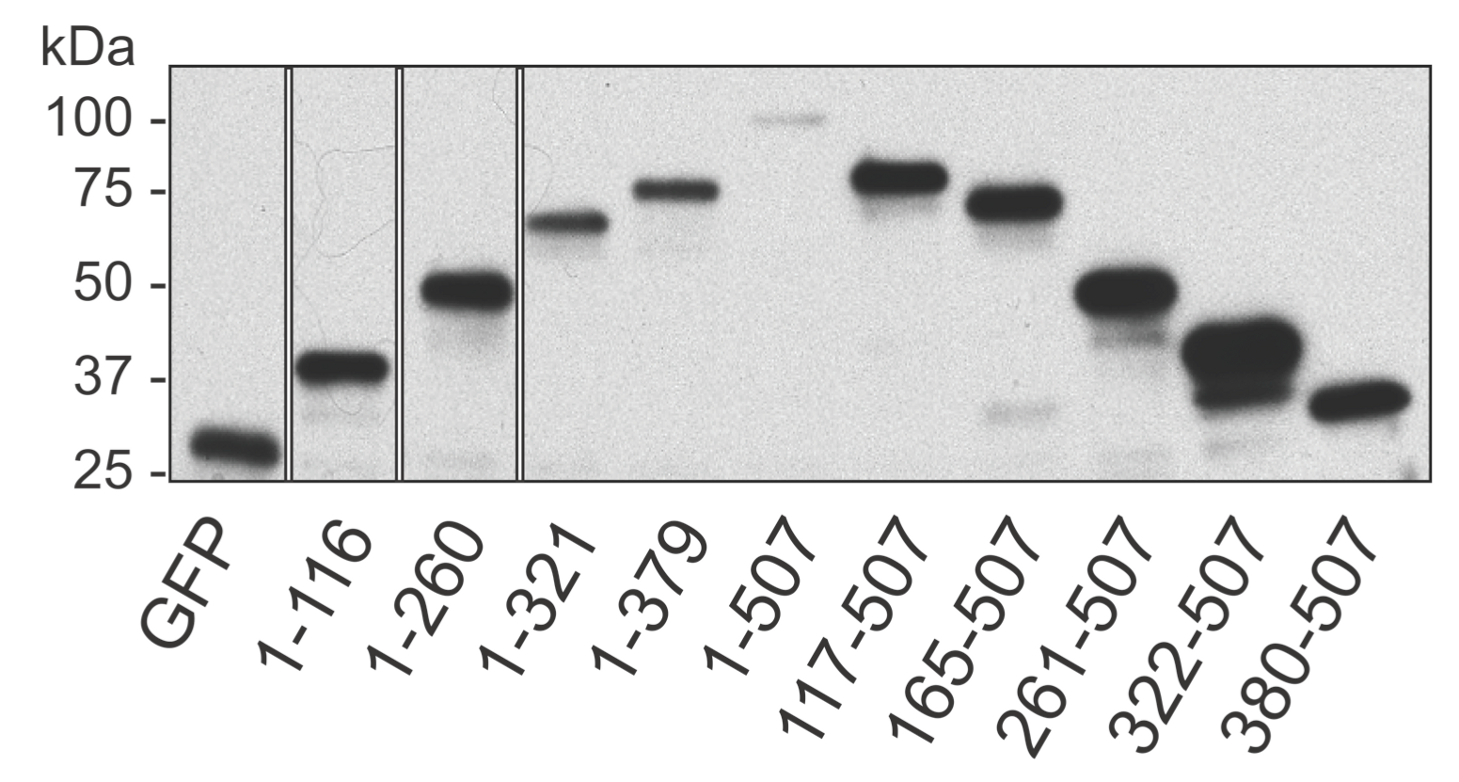

Supplement: Figure S1 — GFP-SidD variants are stably produced in COS1 cells. Transiently transfected COS1 cells producing GFP-SidD fragments were harvested, resuspended in sample buffer, and proteins were separated by SDS-PAGE followed by Western blot analysis using anti-GFP antibody. (TIF) [file ppat.1003382.s001.tif]

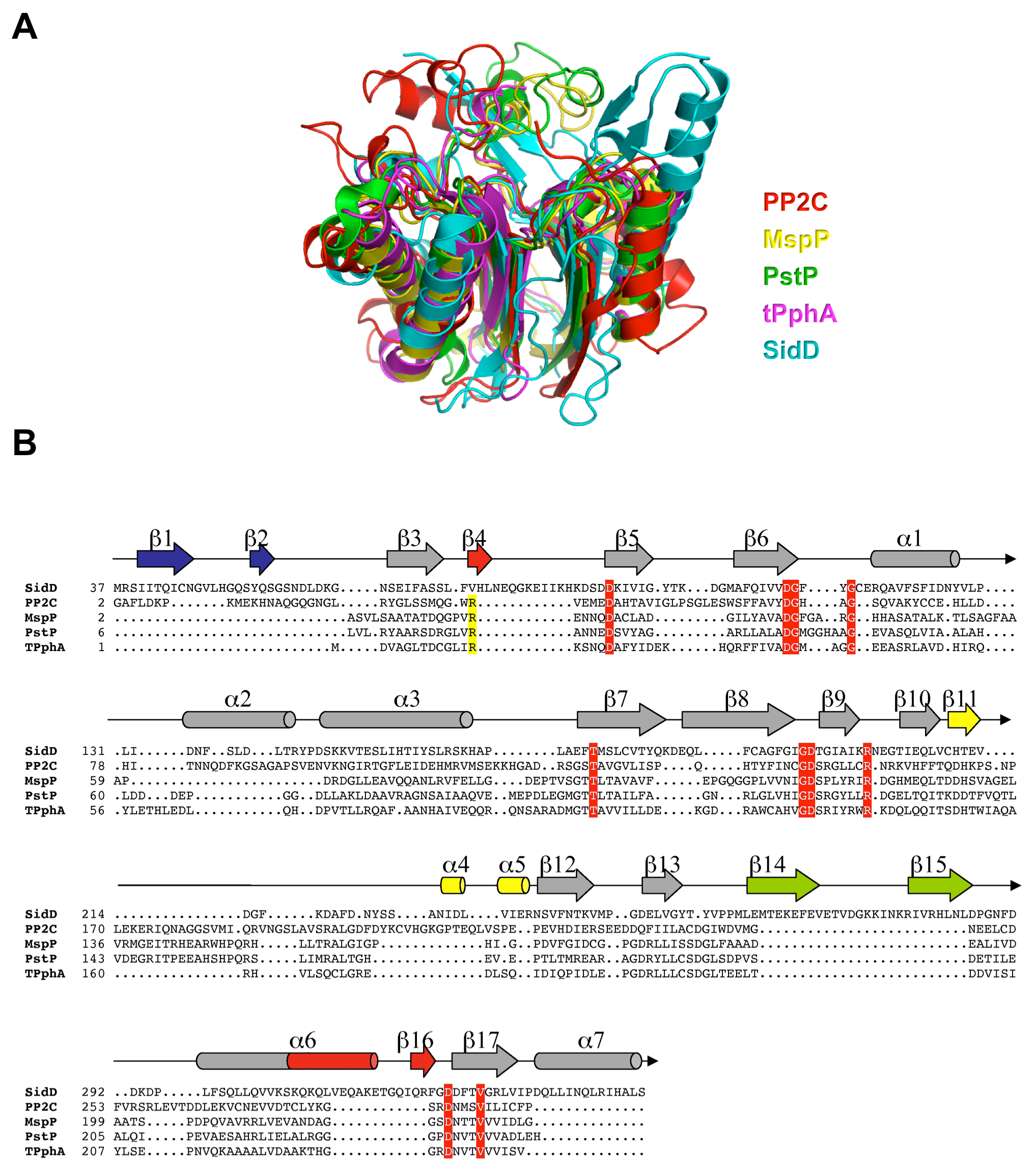

Supplement: Figure S2 — Comparison of SidD with other PPM phosphatases. (A) Superposition of PP2C (phosphatase 2C, PDB 1A6Q) in red, MspP (PPM phosphatase from Mycobacterium smegmatis, PDB 2JFS) in yellow, PstP (PPM phosphatase from Mycobacterium tuberculosis, PDB 1TXO) in green, tPphA (PPM phosphatase from Thermosynechococcus elengatus, PDB 2J82) in pink and SidD in cyan. Domains with no structural homology outside the phosphatase core have been omitted for clarity. (B) Structure-based sequence alignment between the same PPM phosphatases descrived in (A) and SidD. Strictly conserved residues are highlighted in red. Conserved arginine residues, considered to be important for binding the phosphate monoester group during the catalysis are highlighted in yellow. Structural differences present in SidD are colored on the upper diagram as in Fig. 2 A,B. (TIF) [file ppat.1003382.s002.tif]

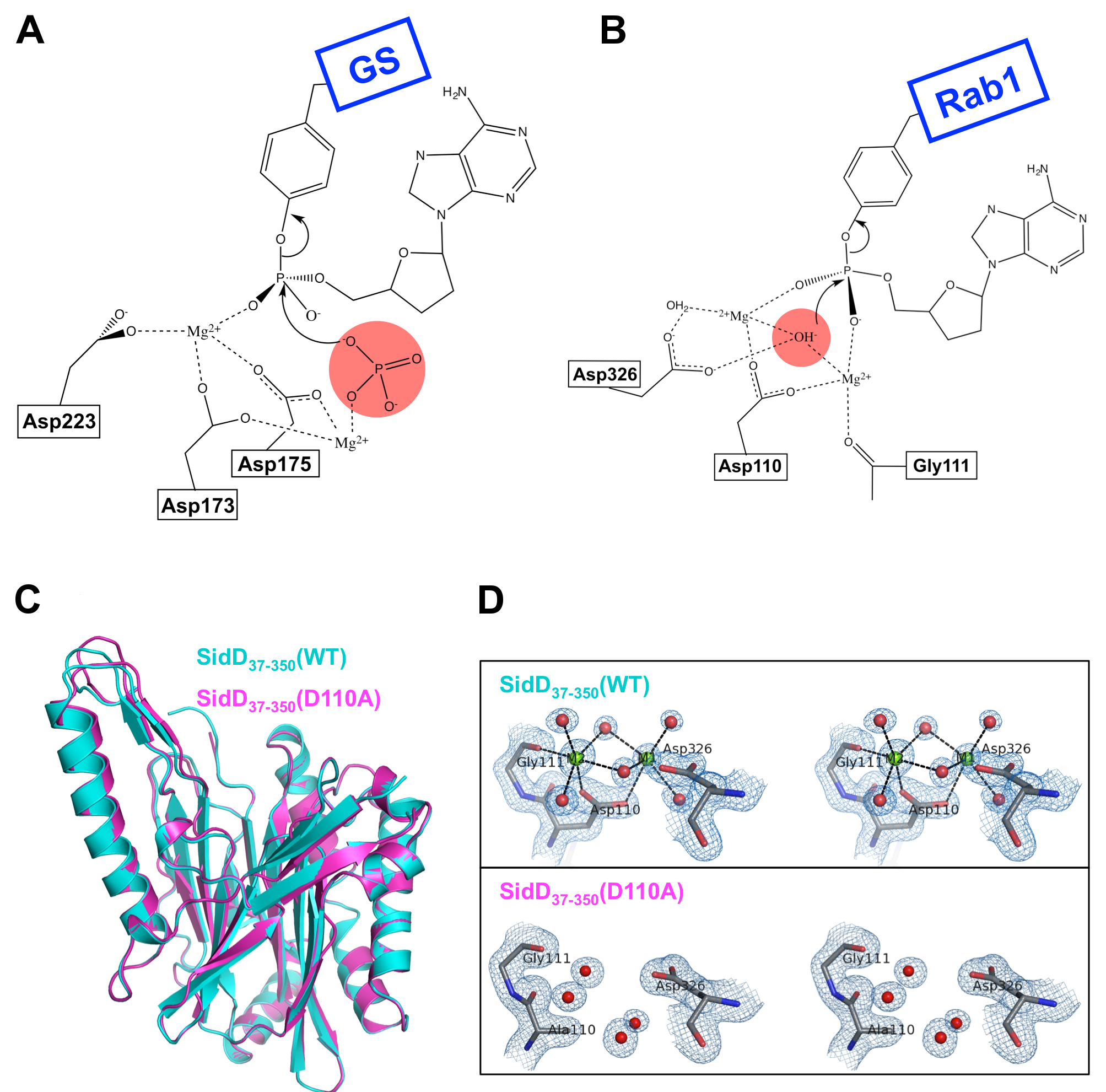

Supplement: Figure S3 — The catalytic center of SidD. (A) Schematic presentation of the mechanism proposed for AT-N based on the reverse reaction catalyzed by some nucleotidyl transferases such as DNA polymerase β and poly(A) polymerase. (B) Schematic view illustrating the catalytic mechanism of SidD adapted from the general mechanism proposed for the PPM phosphatase MspP. (C) Superposition of the structures of wild-type SidD in cyan vs D110A mutant in pink color. (D) Difference electron density map (2Fo-Fc contoured at 1.5σ, blue mesh) of the catalytic site of the wild-type SidD in stereoview representation (upper panel) and the same view of the SidD(D110A) mutant showing the absence of coordinated ions (lower panel). (TIF) [file ppat.1003382.s003.tif]

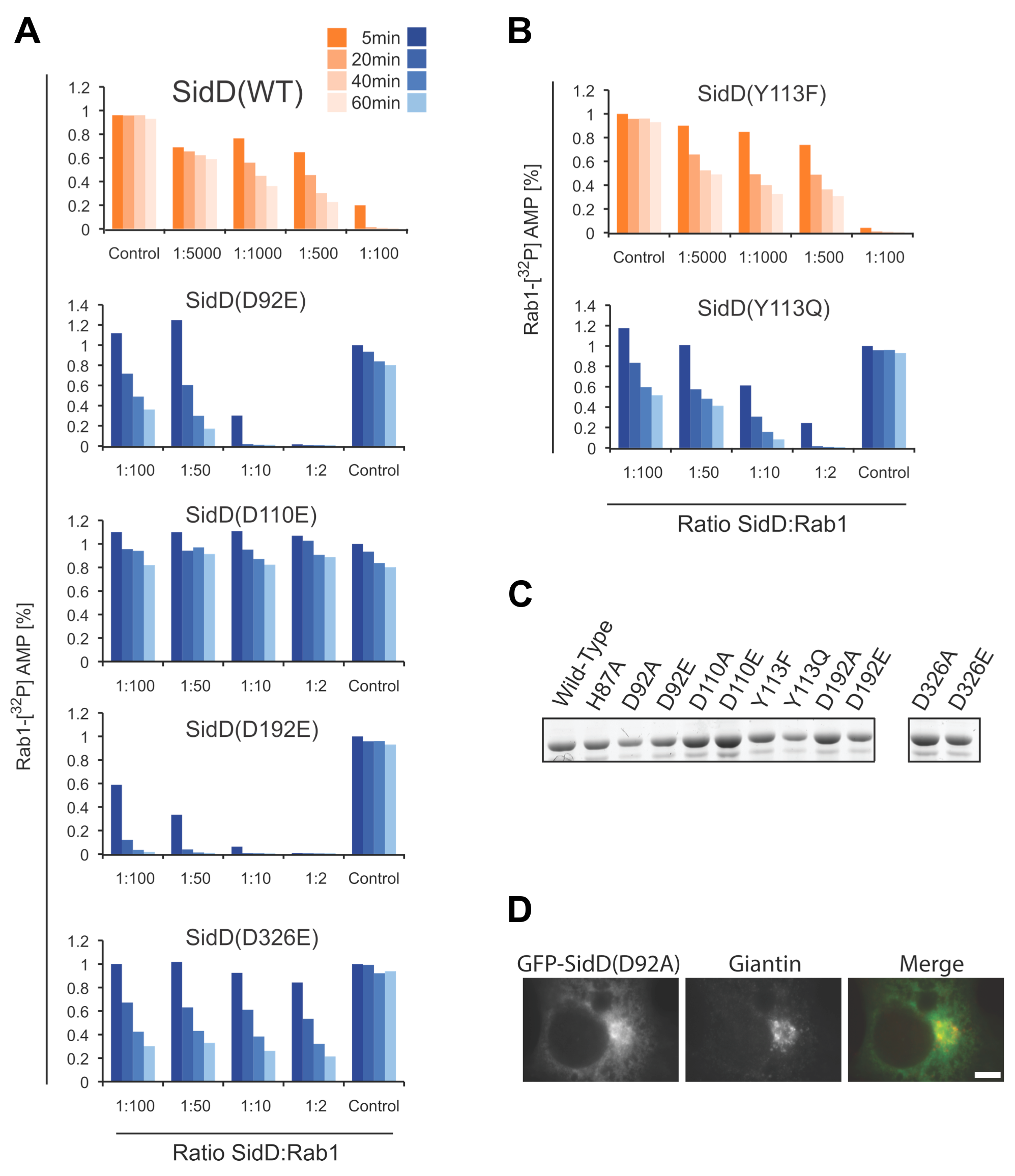

Supplement: Figure S4 — Functional analysis of SidD mutants. Continuation of the de-AMPylation experiment shown in Fig. 5A showing the in vitro de-AMPylation activity of SidD mutants. (A) Analysis of mutants in which aspartate residues at position 92, 110, 192, or 326 have been replaced with similarly charged glutamate. (B) SidD mutants where Y113 has been substituted with either glutamine or the structurally similar phenylalanine. (C) Analysis of SidD mutant protein stability and intracellular localization. Mutant proteins with N-terminal GST-tag purified from E. coli were analyzed by SDS-PAGE and Coomassie staining. (D) Localization of GFP-tagged SidD(D92A) within transiently transfected COS1 cells. SidD mutant proteins showed intracellular localization reminiscent of the wild type protein (Fig. 1C main text), suggesting that their failure to rescue SidM-induced rounding of COS1 cell was not due to a defect in proper targeting of the protein. (TIF) [file ppat.1003382.s004.tif]

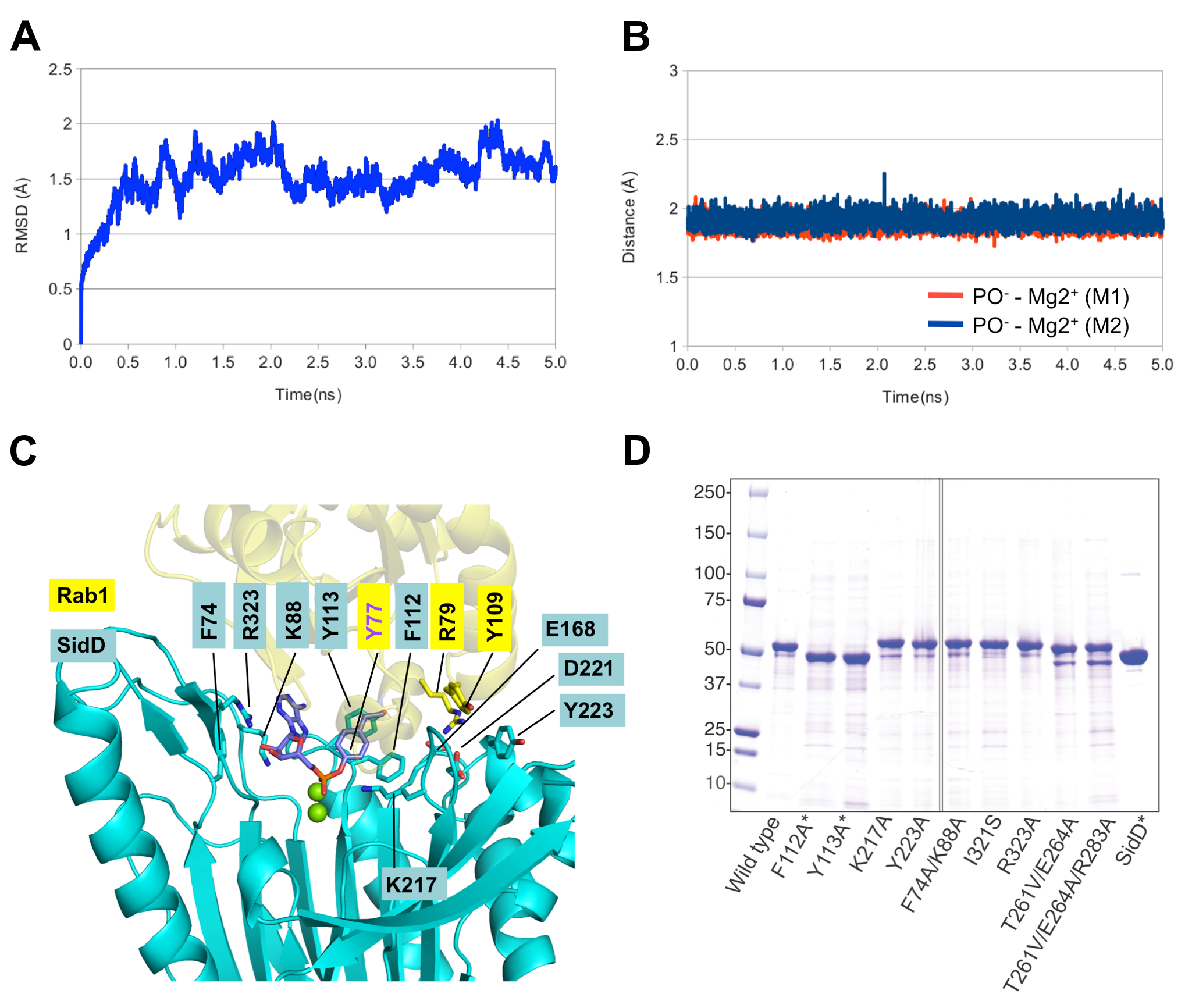

Supplement: Figure S5 — Analysis of the SidD-Rab1 interface. (A) Plot of root mean square deviation (RMSD) of SidD in complex with AMPylated Rab1. (B) Plot of the distances between the non-bridging phosphoryl oxygens of the AMP and the Mg2+(1) and Mg2+(2) catalytic ions of SidD. Note the stable distances over the simulation period. (C) Close-up view of the binding interface residues between SidD and Rab1 from the average structure from the last nanosecond of the MD simulation. (D) Mutant proteins with N-terminal GST-tag purified from E.coli were analyzed by SDS-PAGE and Coomassie staining for their stability and solubility. *: SidD(37-507) variants. (TIF) [file ppat.1003382.s005.tif]

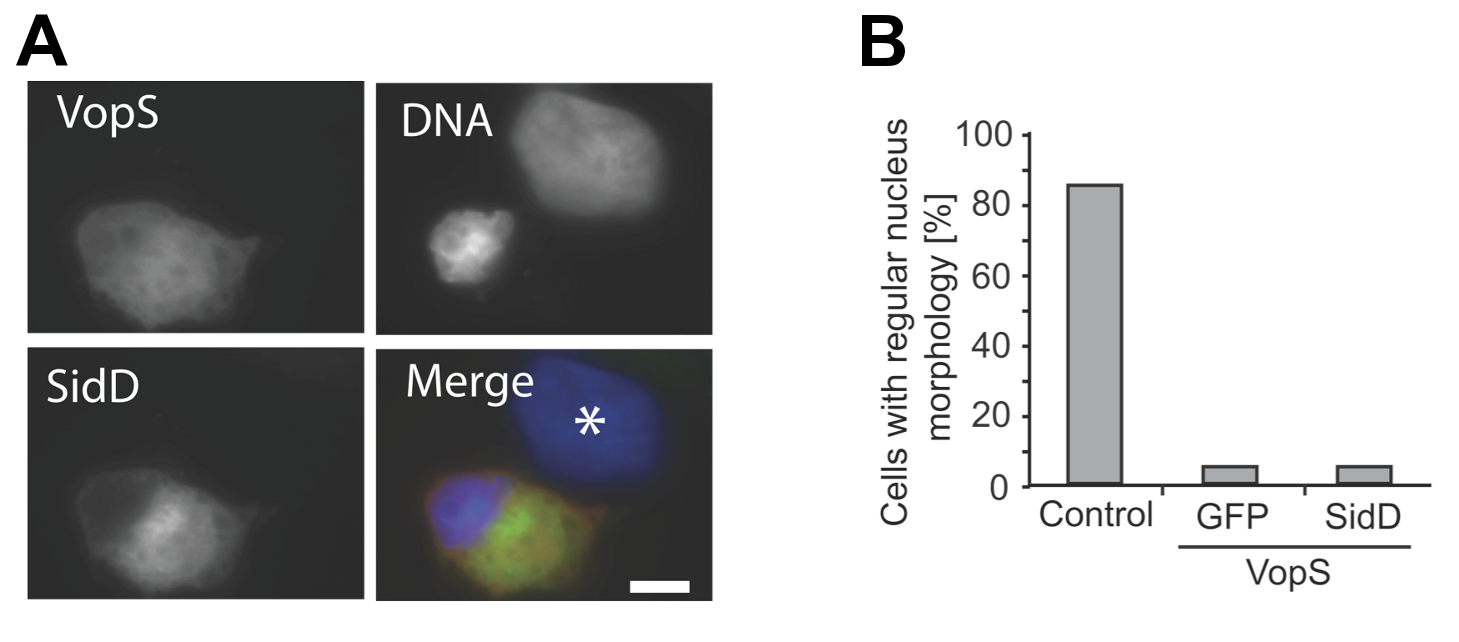

Supplement: Figure S6 — Functional specificity of SidD. (A) VopS-induced cytotoxicity is not rescued by SidD. COS1 cells co-transfected with plasmids encoding mCherry-VopS and GFP-SidD were fixed after 12 hours, nuclei were labeled using Hoechst stain, and nuclear morphology of doubly-transfected and untransfected (*) cells was determined by fluorescence microscopy. Scale bar, 1 µm. (B) Quantification of the experiment shown in (A) showing the percentage of cells with regular nuclear morphology. COS1 cells overproducing plasmid-encoded mCherry-VopS showed extensive cell rounding and nuclear condensation which was not observed in control cells producing mCherry (>90% vs <15%, respectively). The simultaneous presence of either GFP-SidD or GFP alone did not noticeably reduce VopS-induced nuclear condensation, further confirming that Cdc42 was not a substrate for SidD-catalyzed de-AMPylation. Data were obtained from two independent experiments. (TIF) [file ppat.1003382.s006.tif]

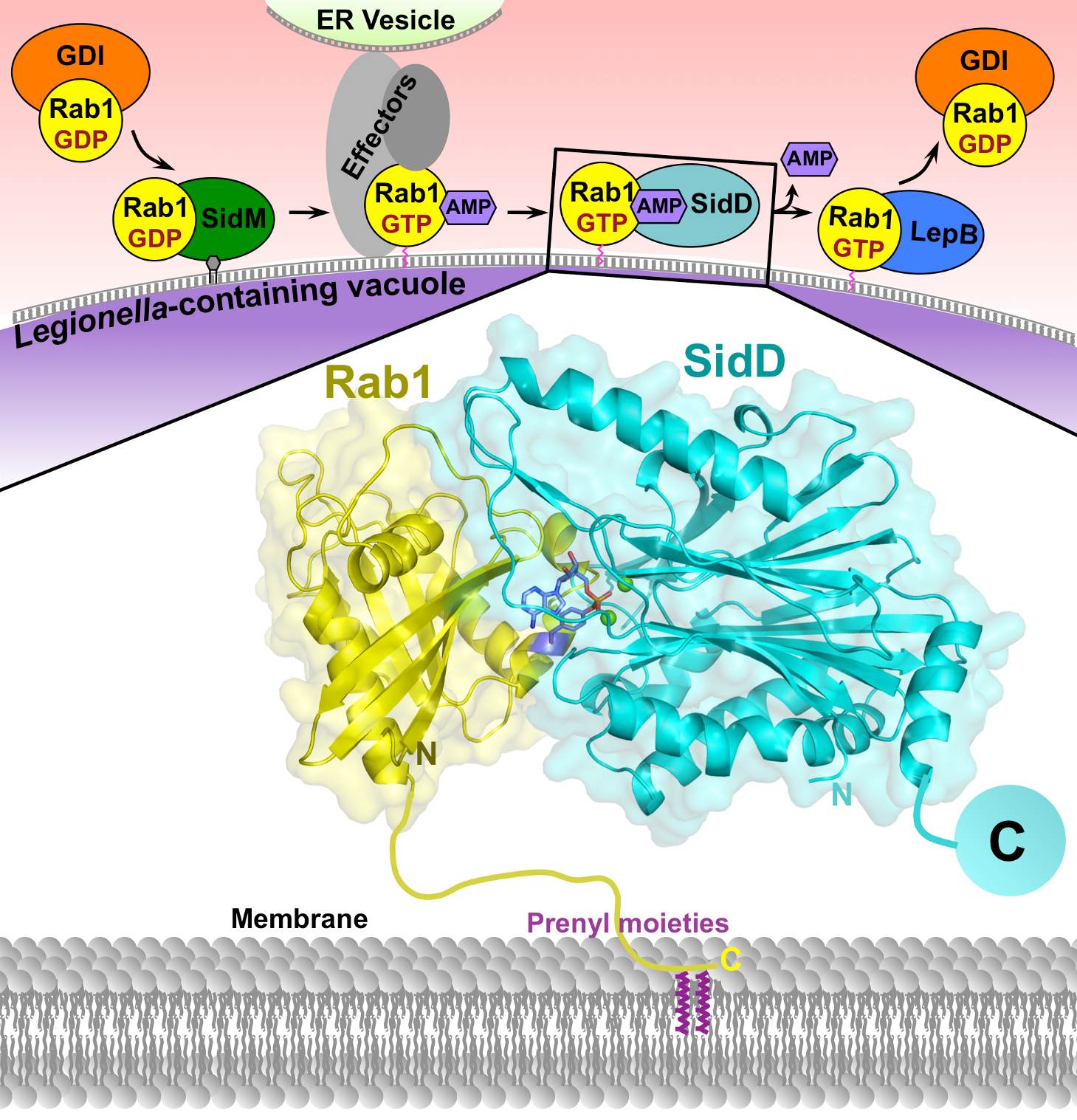

Supplement: Figure S7 — Structural model for the specific recognition of AMPylated Rab1 by SidD. Rab1 is anchored to the LCV membrane through its C-terminal hydrophobic prenyl tails whereas SidD is targeted to the same membrane via its C-terminal domain. Then through complementary shape, charge and hydrophobic interactions the N-terminal domain of SidD binds to AMPylated Rab1 and catalyzes the hydrolysis of the phosphodiester bond between AMP and Tyr77. The configuration of the complex shows how the prenylation anchor of Rab1 and the C-terminal targeting region of SidD are oriented towards the LCV membrane. Yellow, Rab1 in ribbon backbone representation with transparent surface; cyan, SidD in ribbon backbone representation with transparent surface; violet, Tyr77-AMP in stick representation; green spheres, Mg2+ ions; Lilac, prenyl groups. (TIF) [file ppat.1003382.s007.tif]
