# Supplementary material for: Structural Basis for Rab1 De-AMPylation by the Legionella pneumophila Effector SidD
Source: PLoS Pathog. 2013 May 16;9(5):e1003382. doi: 10.1371/journal.ppat.1003382 (PMC3656104; doi:10.1371/journal.ppat.1003382)
Supplement: Text S1 — Additional details of methods used for protein production, purification, X-ray data collection, quantitative elemental analysts, SidD-Rab1 model docking, and refinement by molecular dynamics. (DOCX) [file ppat.1003382.s008.docx]

**SUpporting information**

**Structural basis for Rab1 de-AMPylation by the *Legionella pneumophila* effector SidD**

Yang Chen^1,5,^*, Igor Tascón^2,^*, M. Ramona Neunuebel^1^, Chiara Pallara^3^, Jacqueline Brady^1^, Lisa N. Kinch^4^, Juan Fernández-Recio^3^, Adriana L. Rojas^2^, Matthias P. Machner^1,§^, and Aitor Hierro^2,6,§^

^1^Cell Biology and Metabolism Program, *Eunice Kennedy Shriver* National Institute of Child Health and Human Development, National Institutes of Health, Bethesda, Maryland 20892, USA;

^2^Structural Biology Unit, CIC bioGUNE, Bizkaia Technology Park, 48160 Derio, Spain;

^3^Joint BSC-IRB research program in Computational Biology, Barcelona Supercomputing Center, Barcelona, 08034, Spain;

^4^University of Texas Southwestern Medical Center, Dallas, Texas 75390, USA;

^5^Present address: Health Science Center, Peking University, Beijing 100191, China;

^6^IKERBASQUE, Basque Foundation for Science, 48011 Bilbao, Spain.

*These authors contributed equally to this work

§To whom correspondence should be addressed:

ahierro@cicbiogune.es or machnerm@mail.nih.gov

Short Title: Structure-function analysis of *L. pneumophila* SidD

**SUPPLEMENTAL TABLES**

| **Table S1. Data collection and structure refinement parameters** | | | | |
| --- | --- | --- | --- | --- |
|  | Native (HR) | Native (LR) | Gd-Derivate | D110A |
| **Data collection** | | | | |
| Wavelength | 0.900100 | 1.541800 | 1.541800 | 0.900100 |
| Unit cell parameters  *a,b,c* (Å)  α,β,γ (º) | 60.07, 71.56, 88.30  90, 90, 90 | 60.22, 71.68, 88.42  90, 90, 90 | 60.66, 71.86, 87.71  90, 90, 90 | 60.53, 71.42, 88.19 |
| Resolution (Å) | 50-1.60(1.70-1.60) | 50-2.6 (2.75-2.60) | 50-2.8 (2.95-2.80) | 50-1.9 (2.0-1.9) |
| Rmeas (%) | 5.5 (57.5) | 14.1 (37.8) | 14.1 (34.6) | 9.8 (57.4) |
| Completeness (%) | 99.2 (96.4) | 99.2 (95.6) | 97.5 (90.0) | 99.4 (97.6) |
| Multiplicity | 3.8 (3.7) | 3.9 (3.6) | 3.3 (3.1) | 3.8 (3.6) |
| I/σ | 14.2 (2.14) | 8.9 (3.7) | 8.3 (3.8) | 13.1 (3.2) |
| No.Reflections (observed) | 368480 | 88988 | 52362 | 221329 |
| No.Reflections (unique) | 50486 | 22634 | 18018 | 30812 |
| **Structure refinement** | | | | |
| R-factor (%) | 12.8 |  |  | 14.9 |
| R-free (%) | 17.1 |  |  | 19.3 |
| No Reflections  (R-Free) | 47744  (2555) |  |  | 29271  (1541) |
| No. water molecules | 264 |  |  | 238 |
| No. ions | 15 |  |  | 16 |
| No. glycerol molecules | 3 |  |  | 4 |
| Bond lengths (A° ) | 0.026 |  |  | 0.019 |
| Bond angles (deg.) | 1.99 |  |  | 1.884 |

| **Table S2. Computational alanine scanning of SidD interfacial residues** | | |
| --- | --- | --- |
| **Residue** | **ΔΔG** | **Error** |
| PHE74 | 2.2275 | +/- 0.3787 |
| HIE76 | 2.8151 | +/- 1.4790 |
| GLU79 | -0.2489 | +/- 1.1517 |
| HIS87 | 0.9544 | +/- 1.0010 |
| LYS88 | 5.3875 | +/- 1.2647 |
| ASP91 | 24.3284 | +/- 1.3074 |
| ASP110 | 23.3108 | +/- 1.7948 |
| GLY111 | - |  |
| PHE112 | 6.3533 | +/- 0.7804 |
| TYR113 | 4.9638 | +/- 1.7381 |
| GLU168 | 9.4233 | +/- 1.6063 |
| ASP192 | 12.4205 | +/- 1.7152 |
| VAL213 | -0.1959 | +/- 0.1285 |
| ASP214 | 1.119 | +/- 1.2571 |
| GLY215 | - |  |
| PHE216 | 1.9477 | +/- 0.6203 |
| LYS217 | 13.569 | +/- 2.6881 |
| ASP221 | 15.1827 | +/- 3.2852 |
| ASN222 | -0.9583 | +/- 0.8039 |
| TYR223 | 4.1657 | +/- 1.2352 |
| LYS278 | 2.3351 | +/- 3.5134 |
| GLY319 | - |  |
| GLN320 | 3.3706 | +/- 1.5480 |
| ILE321 | 1.2562 | +/- 0.5992 |
| GLN322 | 2.3905 | +/- 1.9840 |
| ARG323 | 16.6963 | +/- 1.9023 |
| GLY325 | - |  |
| ASP326 | 10.2249 | +/- 1.6446 |

**SUPPLEMENTAL MATERIALS AND METHODS**

**Strains, media, and plasmids**

*L. pneumophila* strains were grown and maintained as described [1,2]. Plasmids for production of recombinantly tagged SidD and Rab1 in Escherichia coli and of fluorescently tagged-proteins in tissue culture cells were described before. All constructs were verified by DNA sequencing. Dr. Kim Orth (UT Southwestern) kindly provided the plasmids for the production of GST-VopSΔ30, and Dr. Jack Dixon (UC San Diego) provided the plasmids for GST-Fic1 and GST-Cdc42. Anti-Rab1B antibody was purchased from Santa Cruz Biotechnology. Anti-*Legionella* antibodies were previously described [3]. Anti-GFP antibody was a kind gift of Dr. R. Hedge (University of Cambridge).

**Protein Expression and Purification**

Recombinant HaloTag-Lem3 was overproduced in the Single Step (KRX) competent *E. coli* strain (Promega) according to manufacturer's instructions. Lem3 was purified using the HaloTag® Protein Purification System according to manufacturer instructions. Briefly, cells were harvested and resuspended in protein purification buffer (50 mM HEPES, 150 mM NaCl, pH 7.5) followed by lysis using a Microfluidics M-110P Microfluidizer®. The cell lysate was spun at 25,000×g for 20 minutes and the supernatant was incubated with pre-equilibrated HaloLink^TM^ Resin for 2 hours at 4^o^C. The resin was then washed a total of 3 times using the protein purification buffer and untagged Lem3 was cleaved off the resin using the Tobacco Etch Virus (TEV) protease for 2 hours at 4^o^C. The HisLink^TM^ resin (Promega) was used to remove the TEV protease from the supernatant. Removal of the TEV protease was verified by SDS-PAGE.

All other recombinant proteins were produced in *E. coli* BL21(DE3) (Stratagene) and purified as previously described [3] using TALON® Metal Affinity Resin for His-AnkX, His-SidD full length and point-mutants or Glutathione Sepharose 4B slurry (GE Healthcare) for full length GST-SidD and the truncated versions, GST-VopSΔ30, GST-Fic1, GST-Cdc42, and GST-Rac1.

SidD_37-350_ was purified from *E. coli* BL21 (DE3). Cells were grown in LB medium to OD_600_ of 0.8 at which protein expression was induced with 1mM isopropyl-β-dithiogalactopiranoside (IPTG) at 20 ºC overnight. Harvested cells were resuspended in TBS (150 mM NaCl, 50 mM Tris-HCl, pH 7.4) supplemented with 5 mM imidazole, 10 mM β-mercaptoethanol (BME) and lysed at 4 ºC by high pressure homogenization at 27 Kpsi (Constant System Ltd). All subsequent purification steps were carried out at 4 ºC. Insoluble material was removed by ultracentrifugation and His-tagged proteins were purified by affinity chromatography using 10 ml of Ni-NTA beads (QIAGEN) packed in a gravity column. After extensive washing with the same loading buffer, proteins were eluted by addition of 200mM imidazole and dialyzed against 60 mM NaCl, 10 mM BME and 50 mM Tris-HCl pH8.5 buffer. The sample was then loaded onto an ion exchange chromatography column, HiTrap Q HP 5 ml (GE Healthcare) followed by an isocratic gradient from 0.06 M to 1 M NaCl in 20 column volumes. Selected fractions were then concentrated and loaded onto a HiLoad 16/60 Superdex 200 Gel Filtration column (GE Healthcare) equilibrated in TBS supplemented with 10 mM BME. Finally, fractions containing pure protein were pooled, concentrated to 1 mg/ml and stored at -80 ºC.

**X-ray Data Collection**

A Gadolinium derivative was obtained by the quick soaking approach [4]. Usable derivates were found after 30 seconds of soaking in mother-liquor supplemented with 100 mM GdCl_2_. Native and Gd-derivative crystals were flash-cooled in liquid nitrogen and the initial diffraction data was collected at home source on a MAR345 Image Plate detector mounted on a Bruker X8 Proteum generator. Posterior data collection at higher resolution for native SidD_37-350_ (1.6Å) and the D110A mutant (1.9 Å) was performed on a Pilatus 6M detector (Dectris) on ID-24 beamline at Diamond Light Source (United Kingdom) and PROXIMA1 beamline at SOLEIL Synchrotron (France). All diffraction data sets were processed using XDS program [5].

**Elemental analysis by ICP-OES**

The Mg^2+^ content in SidD_37-350_ and SidD_37-350_ (D110A) was determined by inductively coupled plasma optical emission spectrometry (ICP-OES) carried out on an ICP-OES spectrometer-ACTIVA (HORIBA Jobin Yvon). Purified proteins at two concentrations (5 and 10 mg/ml) were digested in sealed, acid-washed tubes, (800μL protein + 200μL nitric acid) at 150°C for 1 h and diluted to 4 mL with double-distilled water. The Mg^2+^ content in the digestion was determined against Mg^2+^ standards. The dialysis buffer was analyzed in parallel at the same sample dilution for subtracting the background levels.

**Structural model of the Rab1/SidD complex by docking**

The FFT-based docking program Zdock2.1 [6] and the energy-based pyDock scoring scheme [7] cannot consider ions, cofactors and non-standard residues as for example AMPylated tyrosine. Thus, the initial docking was performed with the unmodified Rab1. In this regard, with the purpose of better estimating the electrostatic interaction energy, we added a negative charge of -1.0 to the Tyr77 hydroxyl group of Rab1 in order to mimic the missing AMP phosphate group, and the D110 residue of SidD was mutated to arginine to simulate the positive charges of the missing Mg2+ ions (which were coordinated to D110 in the original X-ray structure). The docking procedure generated a total of 2,000 rigid-body orientations. Among them, one Rab1/SidD docking orientation had the Rab1 Tyr77 hydroxyl O atom and the SidD Mg2+ ions at close distance (5.4 Å).

**Refinement of the docking model by molecular dynamics**

The parameters file for the ADP cofactor and the AMPpylated Tyr77 residue (Tyr-AMP) were prepared with the AMBER module ANTECHAMBER and the complex topology file was generated using LEAP. Since the residues 270-275 are missing in SidD crystal structure, we added N-terminal acetyl and C-terminal N-Me amide capping groups to A269 and V276 to avoid improper terminal charges on them. Before running the molecular dynamics simulation we performed a short minimization and a five-step equilibration protocol on the solvated structure, in the same conditions as previously described for the MoDEL database [8]. Because of the missing 270-275 loop in our structure, we modified the two final steps, running a 20 ps simulation with harmonic positional restraints to the backbone atoms with force constant of 5 kcal/(mol·Å^2^) (instead of 1 kcal/(mol·Å^2^) by default), followed by a 100-ps MD simulation with 5kcal/(mol·Å^2^) positional restraints only on the capped residues, as well as 2 Å distance restrains between the phosphate group of Rab1 Tyr-AMP and each SidD Mg^2+^ ion of the binding site, in order to keep the coordination. Then, we performed 5 ns MD simulation in isothermal-isobaric ensemble, setting pressure to 1 atm and temperature to 300K, keeping only the restraints to the capped residues. Root mean square deviation (RMSD) and Tyr-Mg2+ ions distances along the MD trajectory were calculated with the ptraj AMBER tool [9].

**SUPPLEMENTAL REFERENCES**

1. Gabay JE, Blake M, Niles WD, Horwitz MA (1985) Purification of Legionella pneumophila major outer membrane protein and demonstration that it is a porin. J Bacteriol 162: 85-91.

2. Feeley JC, Gibson RJ, Gorman GW, Langford NC, Rasheed JK, et al. (1979) Charcoal-yeast extract agar: primary isolation medium for Legionella pneumophila. J Clin Microbiol 10: 437-441.

3. Neunuebel MR, Chen Y, Gaspar AH, Backlund PS, Jr., Yergey A, et al. (2011) De-AMPylation of the small GTPase Rab1 by the pathogen Legionella pneumophila. Science 333: 453-456.

4. Nagem RA, Ambrosio AL, Rojas AL, Navarro MV, Golubev AM, et al. (2005) Getting the most out of X-ray home sources. Acta Crystallogr D Biol Crystallogr 61: 1022-1030.

5. Kabsch W (2010) Xds. Acta Crystallogr D Biol Crystallogr 66: 125-132.

6. Chen R, Weng Z (2003) A novel shape complementarity scoring function for protein-protein docking. Proteins 51: 397-408.

7. Cheng TM, Blundell TL, Fernandez-Recio J (2007) pyDock: electrostatics and desolvation for effective scoring of rigid-body protein-protein docking. Proteins 68: 503-515.

8. Meyer T, D'Abramo M, Hospital A, Rueda M, Ferrer-Costa C, et al. (2010) MoDEL (Molecular Dynamics Extended Library): a database of atomistic molecular dynamics trajectories. Structure 18: 1399-1409.

9. Case DA, Cheatham TE, 3rd, Darden T, Gohlke H, Luo R, et al. (2005) The Amber biomolecular simulation programs. J Comput Chem 26: 1668-1688.
